# Preparation and Diels–Alder/cross coupling reactions of a 2-diethanolaminoboron-substituted 1,3-diene

**DOI:** 10.3762/bjoc.5.45

**Published:** 2009-09-21

**Authors:** Liqiong Wang, Cynthia S Day, Marcus W Wright, Mark E Welker

**Affiliations:** 1 Department of Chemistry, Wake Forest University, P.O. Box 7486, Winston-Salem, NC 27109 (USA)

**Keywords:** cross coupling, Diels–Alder, organoboron

## Abstract

A 2-diethanolamine boronyl substituted 1,3-diene has been synthesized in high yield and characterized spectroscopically as well as by X-ray crystallography. This diene has then subsequently been used in a number of fast, high yielding Diels–Alder/cross coupling reactions.

## Introduction

Our group [1] and the Tada group [2] independently reported the preparation and Diels–Alder reactions of pyridine cobaloxime dienyl complexes over 15 years ago. Since that time, we have reported a number of synthetic routes to these and other related types of cobalt dienyl complexes as well as their subsequent cycloaddition and demetallation chemistry [3–5], and other groups have now made use of the cycloadducts thus prepared [6] as well as the methodology [7].

We have now subsequently reported the preparation of 2-BF_3_ substituted 1,3-butadienes and demonstrated that they can be used in sequential Diels–Alder/cross coupling reactions [8–9]. These trifluoroborate substituted dienes are stable but their organic solvent solubility is not ideal. Preparation of more highly substituted BF_3_ dienes also requires a transmetallation protocol which yields a Grignard-BF_3_ by-product which has to be separated from the desired diene [9]. To overcome these methodology challenges we have begun to prepare diethanolaminoboron substituted dienes and we communicate our first results in this area here.

## Results and Discussion

**Scheme 1:**
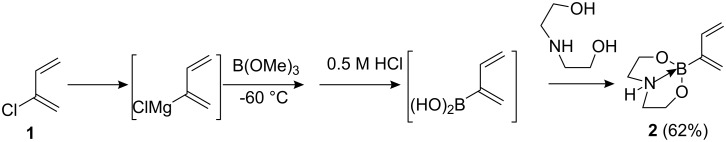
Synthesis of 2-diethanolaminoborate-1,3-butadiene.

**Figure 1:**
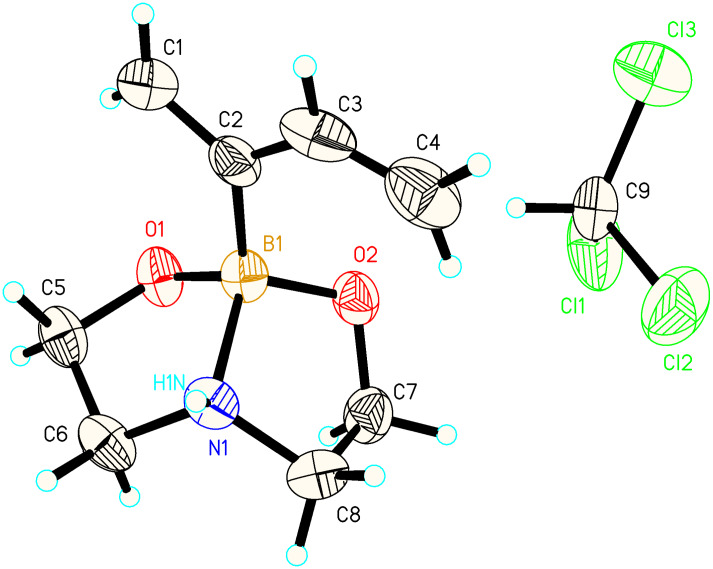
Molecular structure of boron substituted diene **2**.

The diethanolamine boronyl substituted diene **2** was obtained as white needles on a several gram scale from a simple procedure which involved preparing the Grignard reagent from chloroprene **1**, adding this reagent to trimethoxyborane followed by the addition of dilute HCl and diethanolamine (Scheme 1). The boron substituted diene **2** thus obtained has C1 (δ 5.23 vs δ 5.04, 4.96 (d_6_-DMSO) and C3 (δ 6.31 vs. δ 6.19) hydrogen atoms which are significantly more deshielded than the BF_3_ substituted diene. In the solid state (Figure 1, see Supporting Information), C(1)–C(2) and C(2)–C(3) bond lengths were virtually identical in both dienes whereas B–C(2) (1.609(5) Å vs 1.576(13) Å) and C(3)–C(4) (1.308(6) Å vs 1.279(13) Å) were significantly longer in the diethanolamine boronyl diene **2**.

**Scheme 2:**
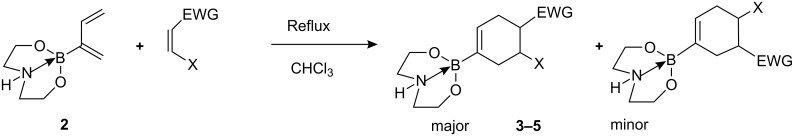
Diels–Alder reactions.

This diethanolamine boronyl diene **2** has proved to be significantly more reactive and more regioselective in Diels–Alder reactions compared to its BF_3_ diene counterpart (Table 1, Scheme 2) [8]. Qualitatively, we initially noticed that whereas the BF_3_ diene required 16 h of heating at 95–100 °C in a sealed tube in toluene with N-phenylmaleimide to obtain >90% yield of cycloadduct, the diethanolamine boronyl diene **2** reacted with this same dienophile to afford a 98% isolated yield of cyclaooduct **4** after only 15 min at 25 °C! We tried to get more quantitative rate constant data about this Diels–Alder reaction via NMR spectroscopy but when we try to perform this reaction under pseudo first order conditions at −10 °C, we can only say that the t_1/2_ is less than 4 minutes. Attempts to get more accurate kinetic data by NMR at −40 °C resulted instead in diene **2** precipitation. This diene **2** is by far the most reactive main group element substituted diene we have made in the boron or silicon substituted series to date. What is perhaps even more surprising to us is that this diene **2** is even more reactive than the most reactive cobaloxime substituted diene we ever prepared in our earlier work [10] and those cobaloxime dienes consistently favored the *s-cis* conformation in the solid state. Diene **2** is in the *s-trans* conformation in the solid state (Figure 1) but in this case we suspect that the preference for the *s-trans* conformer is due to intermolecular hydrogen bonding between the N–H and one of the adjacent molecule’s boronate oxygen atoms. This hydrogen bonding would make a C(2)–C(3) dihedral angle of 50–60° (on the order of those we observed in cobaloxime diene solid state structures) unfavorable. At 25 °C in CDCl_3_, we saw no evidence for the *s-cis* conformer by NOESY.

**Table 1:** Diels–Alder reactions of diene **2**.

Product #	Dienophile	Temp (°C)	Time (h)	Yield (%)	Major:Minor

**3**	ethyl acrylate	reflux	6	84	16.4:1
**4**	*N*-phenyl maleimide	25	0.25	98	NA
**5**	2-methyl *N*-phenyl maleimide	reflux	8	95	4.0:1

In an effort to further understand this reactivity difference, geometry-optimization with DFT using the B3LYP functional and a 6-31G(d) basis set followed by population analysis was performed using Gaussian 03 on 2-diethanolaminoboronyl-1,3-butadiene (**2**) and its BF_3_ diene counterpart. 2-Diethanolaminoboronyl-1,3-butadiene has a HOMO energy of −6.00 eV, whereas its BF_3_ diene counterpart has a HOMO energy of −12.58 eV. These energies are consistent with our observations that 2-diethanolaminoboronyl-1,3-butadiene (**2**) is more reactive than its BF_3_ diene counterpart. Furthermore, a Mulliken population analysis indicates a build-up of electron density on carbons C1 and C4 of 0.15e and 0.14e respectively in 2-diethanolaminoboronyl-1,3-butadiene (**2**) compared to its BF_3_ diene counterpart, which is also consistent with our observations. In addition to enhanced Diels–Alder reaction rates, we also noted greatly improved regioselectivities (Table 1). Whereas the BF_3_ diene required 36 h of heating to 95–100 °C in a sealed tube in ethanol to provide a 3.3:1 mixture of regioisomers from reaction with ethyl acrylate, the diethanolamine boronyl diene **2** reacted with this same dienophile at reflux for 6 h to provide a 16.4:1 mixture of para (1,4) to meta (1,3) isomers **3** in identical isolated yield. Similarly, when we used a citraconamide derivative (2-methyl *N*-phenylmaleimide), we isolated cycloadduct **5** in high yield although with reduced regioselectivity (4:1). However, the BF_3_ diene proved unreactive with citraconic acid derived dienophiles. This diethanolamine boronyl diene **2** once again reacted under much milder conditions and with better regioselectivity than highly reactive silicon substituted dienes we have also reported previously [11].

**Scheme 3:**
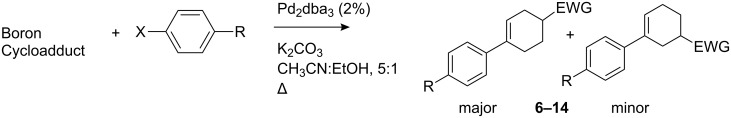
Cross coupling reactions.

Lastly, in order to prove that diethanolamine boronyl diene **2** could serve as a synthon for a host of other organic dienes, we took cycloadducts **3**–**5** and proved that they could be cross coupled efficiently to iodobenzene, 4-trifluoromethyl-1-iodobenzene, and 4-iodoanisole (Table 2, Scheme 3). Cross coupled cycloadducts **6**–**14** were all isolated in good to excellent yield and regioselectivities observed in the original Diels–Alder reactions were maintained after cross coupling.

**Table 2:** Results of cross coupling reactions.

Entry	Cycloadduct (#)	R	Yield (%)	Isomer ratio	Product (#)

1	**3**	H	85	17.2:1	**6**
2	**3**	CF_3_	97	17.9:1	**7**
3	**3**	OMe	80	18.0:1	**8**
4	**4**	H	64	NA	**9**
5	**4**	CF_3_	70	NA	**10**
6	**4**	OMe	60	NA	**11**
7	**5**	H	58	3.5:1	**12**
8	**5**	CF_3_	70	3.4:1	**13**
9	**5**	OMe	75	3.3:1	**14**

## Conclusion

In conclusion, we report a simple preparation of a 2-boronyl substituted 1,3 diene which has proved to be the most reactive 2-main group element or 2-transition metal element substituted diene for Diels–Alder reactions that we have prepared to date. We have also demonstrated that this boron-substituted diene can serve as a synthon for a host of organic dienes via cross coupling reactions which we performed on Diels–Alder reaction cycloadducts.

## Experimental

**Preparation of 1,3-butadiene-2-diethanolamine boronate 2**: A mixture of magnesium (1.0 g, 41.1 mmol), 1,2-dibromoethane (0.5 mL), and THF (10 mL) was refluxed under nitrogen for 15 min to activate the magnesium. To the mixture anhydrous zinc chloride (0.6 g) in THF (60 mL) was added and reflux was continued for another 15 min. 2-Chloro-1,3-butadiene (4.9 mL, 25 mmol) (density 0.915 g/mL, 50% in xylene) and 1,2-dibromoethane (0.95 g, 5 mmol) in THF (30 mL) were added dropwise over a period of 30 min. This addition was controlled so as to bring the mixture into a gentle reflux. The color of the contents changed gradually from grayish white to greenish black. The mixture was heated to reflux for an additional 30 min after completion of the addition. The Grignard reagent thus obtained was immediately added dropwise to a solution of trimethoxyborane (4.25 mL, 38.5 mmol) in THF (25 mL) using a double-ended needle. The addition was controlled in such a way that the internal temperature of the mixture was maintained below –60 °C all the time. After completion of the addition, the solution was allowed to warm to room temperature quickly. The cloudy gray colored reaction mixture was stirred for 1 h. To the resulting mixture at room temperature, 0.5 M HCl solution (100 mL) was added. The reaction mixture was extracted with Et_2_O (2 × 75 mL). The combined colorless clear organic layers were dried over MgSO_4_, and the volatiles were removed by a rotary evaporator (30 °C, 20 Torr) to yield the dieneboronic acid. The boronic acid was added at once to a solution of diethanolamine (0.8 equiv, 22.5 mmol, 8.411g) dissolved in THF (100 mL). Sodium sulfate (8 g) was added and refluxed for 6 h. At the end of the reaction, the flask was cooled to room temperature. Solid Na_2_SO_4_ was separated from the solution by filtration. The solution was reduced by 50 mL using a rotary evaporator. A cold bath of −30 °C was used to induce crystallization. After 4 h, the solid was filtered and washed with cold chloroform. The product **2** was obtained as white needles (2.40 g, 14.4 mmol, 62.4%). ^1^H NMR (300 MHz, CDCl_3_) δ 6.51 (dd, *J* = 17.9, 10.9 Hz, 1H-H3), 5.46–5.40 (m, 3H), 4.98 (dd, *J* = 17.9, 1.9 Hz, 1H-H4), 5.18 (s, 1H-H7), 4.05 (m, 2H-H5,8), 3.89 (m, 2H-H5,8), 3.31 (m, 2H-H6,9), 2.76 (m, 2H-H6,9) ^13^C NMR (300, MHz, CDCl_3_) δ 143.6-C3, 124.3-C4, 114.6-C1, 63.4-C5,8, 52.1-C6,9, the signal of carbon C2 next to a tetravalent boron is generally not observed due to quadrupolar broadening [12]. Elemental anal. calcd for C_8_H_14_BNO_2_: C, 57.53; H, 8.45. Found: 57.06, 8.44.

### Representative Diels–Alder procedure

**Preparation of Diels–Alder product 3:** Diene **2** (0.167 g, 1 mmol) and ethyl acrylate (0.700 g, 7 mmol) were dissolved in chloroform (15 mL) in a round bottomed flask and refluxed for 6 h. The white product was precipitated with pentane (150 mL) and obtained by vacuum filtration, (0.224 g, 0.84 mmol, 84%). **3**: ^1^H NMR (300 MHz, CDCl_3_) δ 5.91 (m, 1H), 4.86 (s, 1H), 4.12 (q, *J* = 7.25, 2H), 3.97 (m, 2H), 2.893 (m, 2H), 3.224 (m, 2H), 2.79 (m, 2H), 2.48 (m, 1H), 2.23 (m, 2H), 2.11(m, 2H), 1.99 (m, 1H), 1.76 (s, 1H), 1.25 (t, *J* = 7.25, 3H). ^13^C NMR (300 MHz, CDCl_3_) δ **Major isomer:** 176.7, 139.9 (=C-B), 126.9, 62.85, 62.81, 60.0, 51.2, 40.1, 39.8, 28.6, 26.4, 25.9, 14.0. **Minor isomer selected resonances:** 176.2, 127.6, 24.7, 24.6. Major isomer: minor isomer = 16.4:1. Elemental anal. calcd. for C_13_H_22_BNO_4_: C, 58.45; H, 8.30. Found: 58.17, 8.32.

### Representative Suzuki coupling procedure

General procedure: Boron compounds and iodoaromatic compounds were added to a N_2_ flushed flask with Pd_2_(dba)_3_ and K_2_CO_3_ in acetonitrile and ethanol (30 mL). The mixture was refluxed for 36 h and cooled to room temperature. The solution was filtered through silica gel to remove catalysts. The filtrate was quenched with water (50 mL) and extracted with Et_2_O (4 × 50 mL). The combined organic layers were dried over MgSO_4_ and volatiles were removed by rotary evaporation. The resulting cross-coupled cycloadduct residue was purified by flash chromatography (ethyl ether:hexane = 1:1). Optimization of conditions: 2% Pd_2_(dba)_3_ [Tris(dibenzylideneacetone)dipalladium (0)], acetonitrile:ethanol = 5:1, boron cycloadduct:iodoaromatic compounds = 1:2, K_2_CO_3_ (3 equiv) reaction time: 36 h.

**Preparation of 6-(4-methoxyphenyl)-3a-methyl-2-phenyl-3a,4,7,7a-tetrahydro-1*H*-isoindole-1,3(2*H*)-dione (14)**: Following the general procedure, 4-iodoanisole (0.234 g, 1 mmol) and **5** (0.178 g, 0.5 mmol) were added along with Pd_2_(dba)_3_ (10 mg) and K_2_CO_3_ (0.207 g, 1.5 mmol) to a flask under N_2_ (30 mL acetonitrile and ethanol). The flask was heated and refluxed for 36 h. The resulting brown oily crude product mixture was subjected to flash chromatography to yield the cross-coupled product as a white solid (0.134 g, 0.39 mmol, 78%). **14**: ^1^H NMR (300 MHz, CDCl_3_) δ **Major isomer:** 7.38 (d, *J* = 7.5 Hz, 2H), 7.31 (d, *J* = 8.7 Hz, 2H), 7.26 (m, 1H), 7.13 (d, *J* = 7.5 Hz, 2H), 6.85 (d, *J* = 8.8 Hz, 2H), 6.1 (m, 1H), 3.80 (s, 3H), 3.25 (dd, *J* = 15.4, 2.4 Hz, 1H), 2.99 (dd, *J* = 6.5, 2.4 Hz, 1H), 2.86 (dd, *J* = 15.4, 6.5 Hz, 1H), 2.61 (ddt, *J* = 15.4, 6.5, 2.4 Hz, 1H), 2.16 (dd, *J* = 15.4, 2.4 Hz, 1H), 1.50 (s, 3H). **Minor isomer selected resonances**: 3.15 (d, *J* = 15.4), 2.44 (m), 2.30 (m). ^13^C NMR (300 MHz, CDCl_3_) δ 182.3, 178.5, 159.5, 139.8, 133.1, 132.4, 129.4, 128.8, 127.0, 126.8, 122.0, 114.3, 55.6, 48.4, 45.1, 36.7, 30.6, 25.9. Elemental anal. calcd for C_22_H_21_NO_3_: C, 76.06; H, 6.09. Found: 76.34, 6.31.

## Supporting Information

File 1 ^1^H and ^13^C NMR spectra of compounds **2–14**.

File 2 Experimental procedures for compounds **4**–**13**.
