# Supplementary material for: Preparation and Diels–Alder/cross coupling reactions of a 2-diethanolaminoboron-substituted 1,3-diene
Source: Beilstein J Org Chem. 2009 Sep 21;5:45. doi: 10.3762/bjoc.5.45 (PMC2779738; doi:10.3762/bjoc.5.45)

# Supporting Information

**for**

**Preparation and Diels–Alder/cross coupling reactions of a 2-diethanolaminoboron-substituted 1,3-diene**

Liqiong Wang, Cynthia S. Day, Marcus W. Wright and Mark E. Welker*

Address: Department of Chemistry, Wake Forest University, P.O. Box 7486, Winston-Salem, NC 27109 (USA)

Email: Mark E. Welker - welker@wfu.edu

^1^H and ^13^C NMR spectra of compounds **2–14**


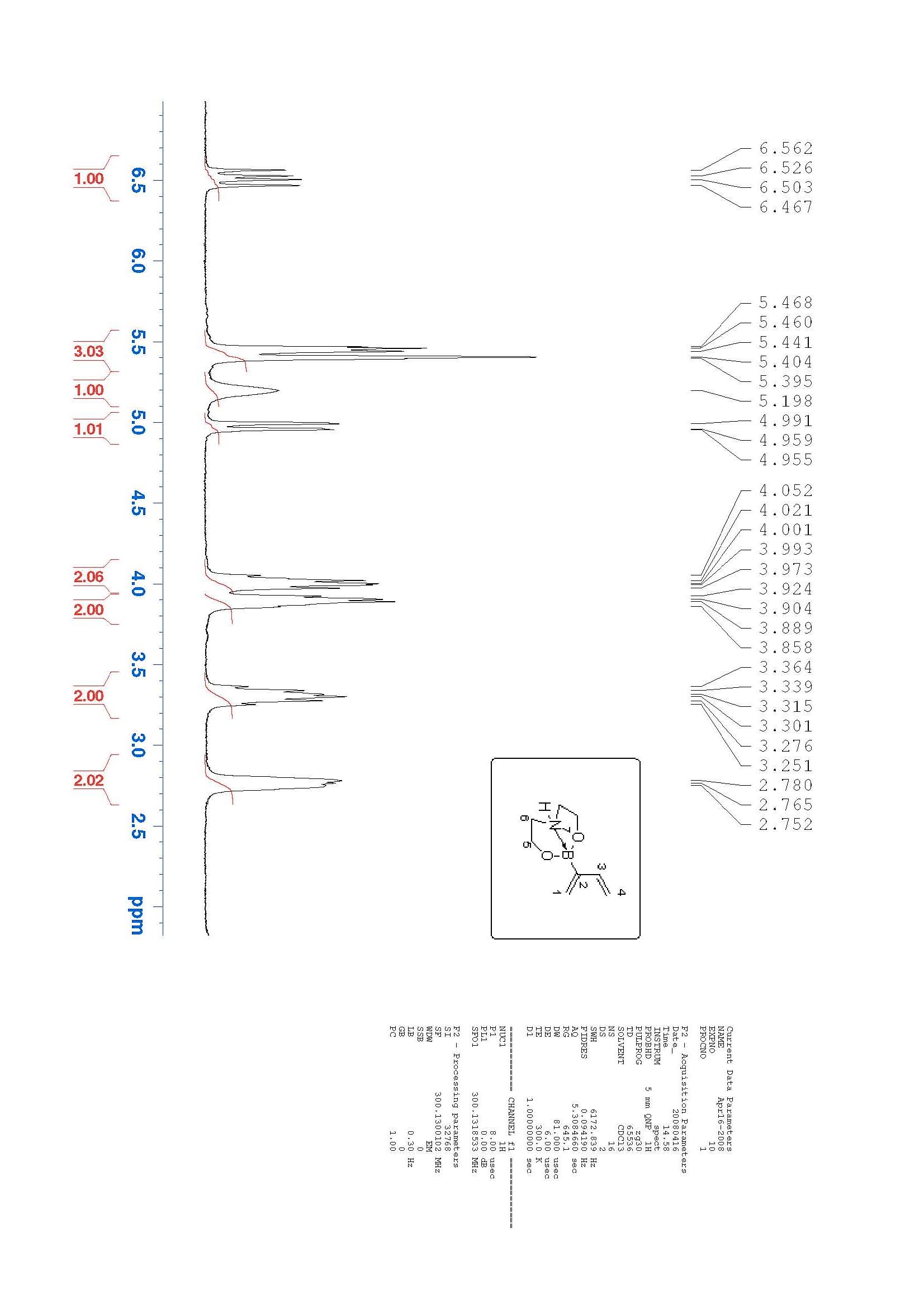


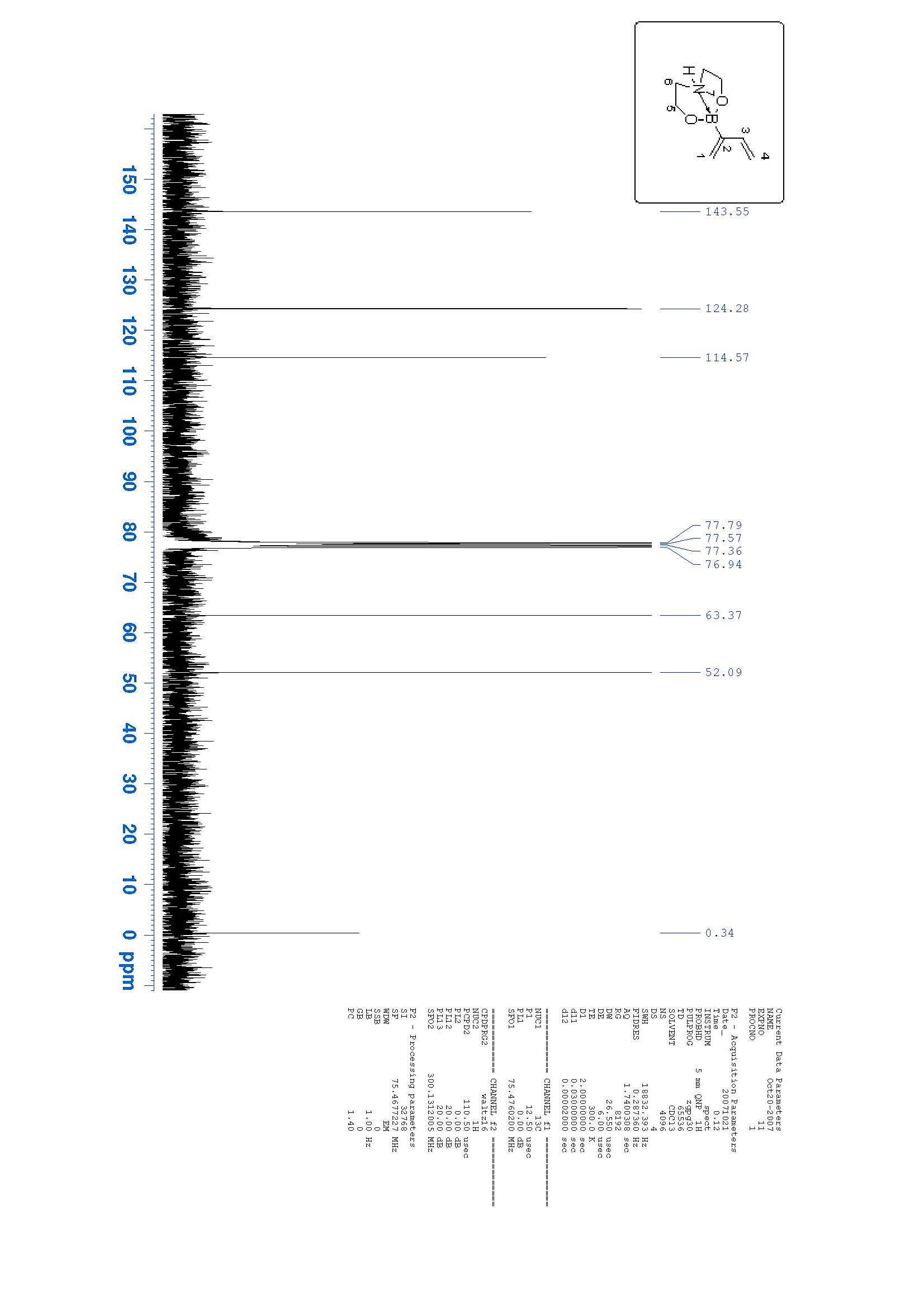


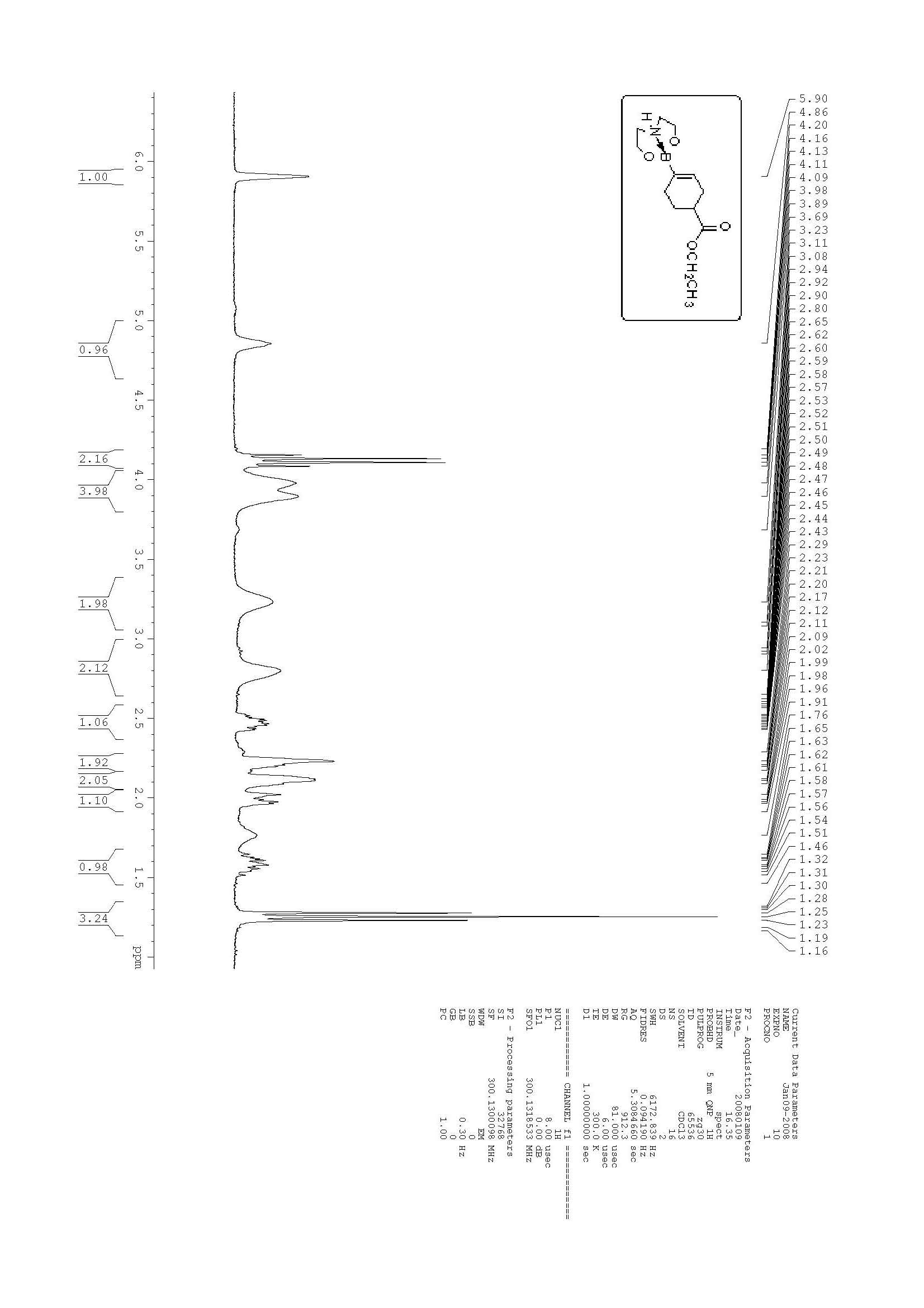


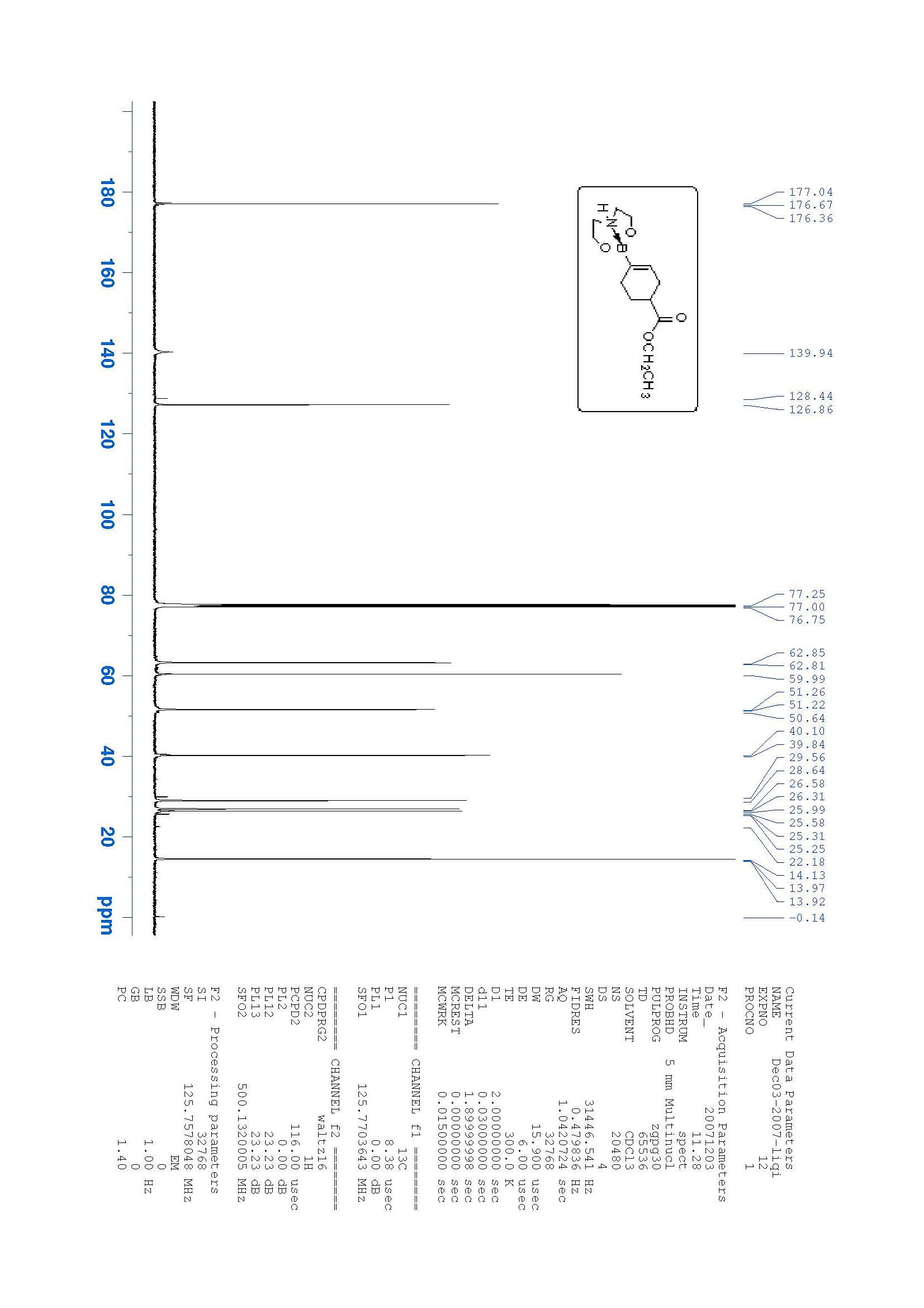


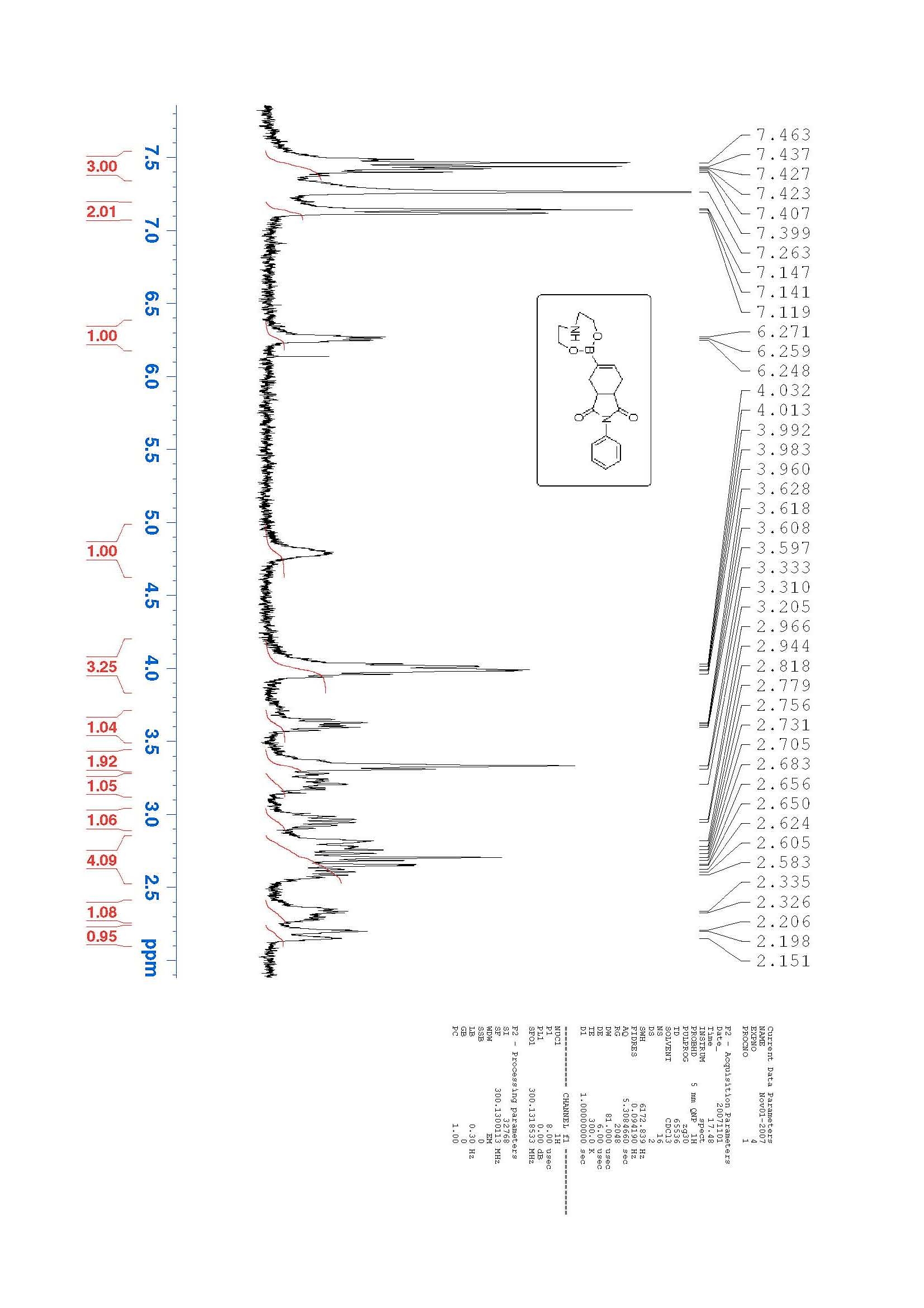


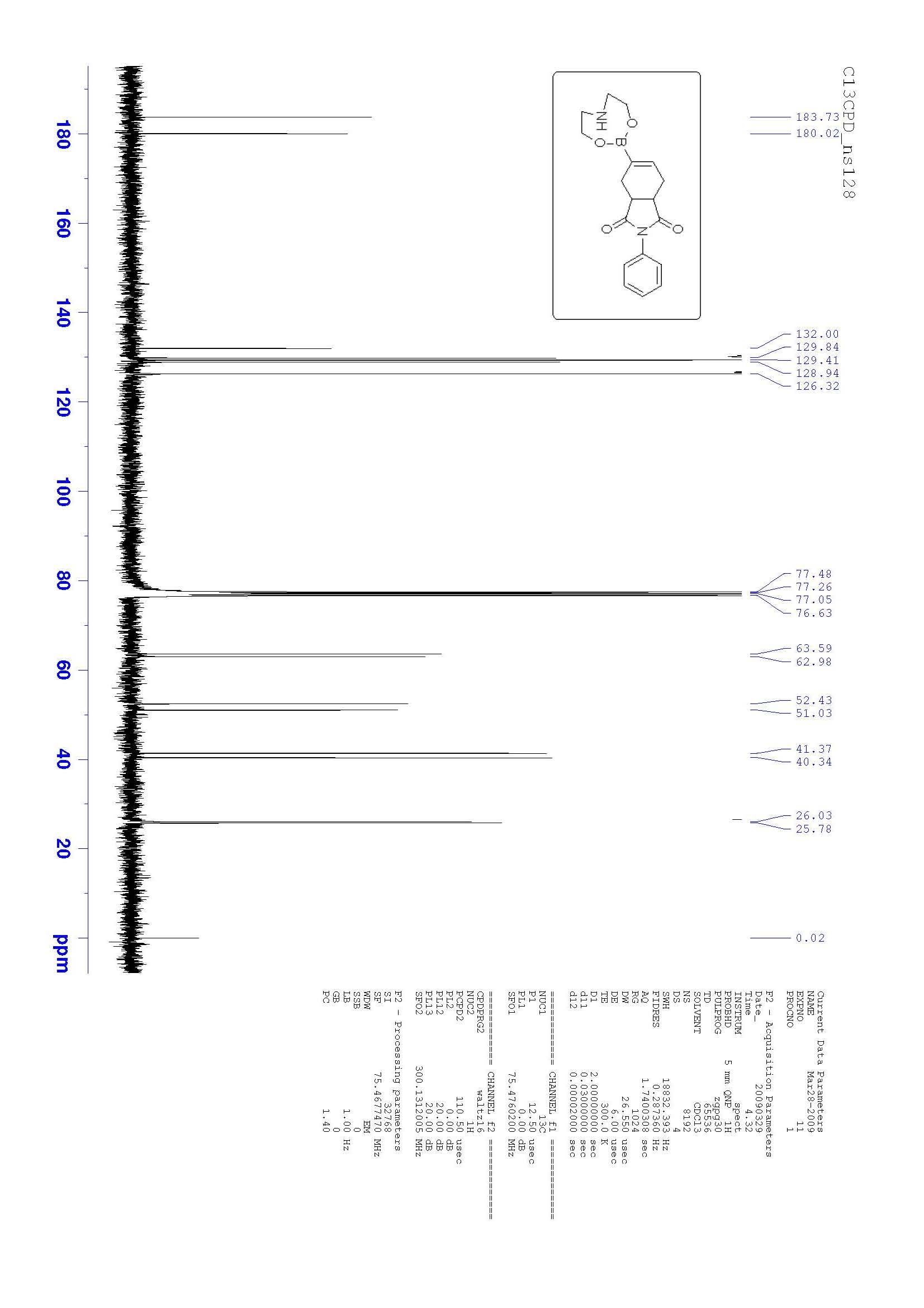


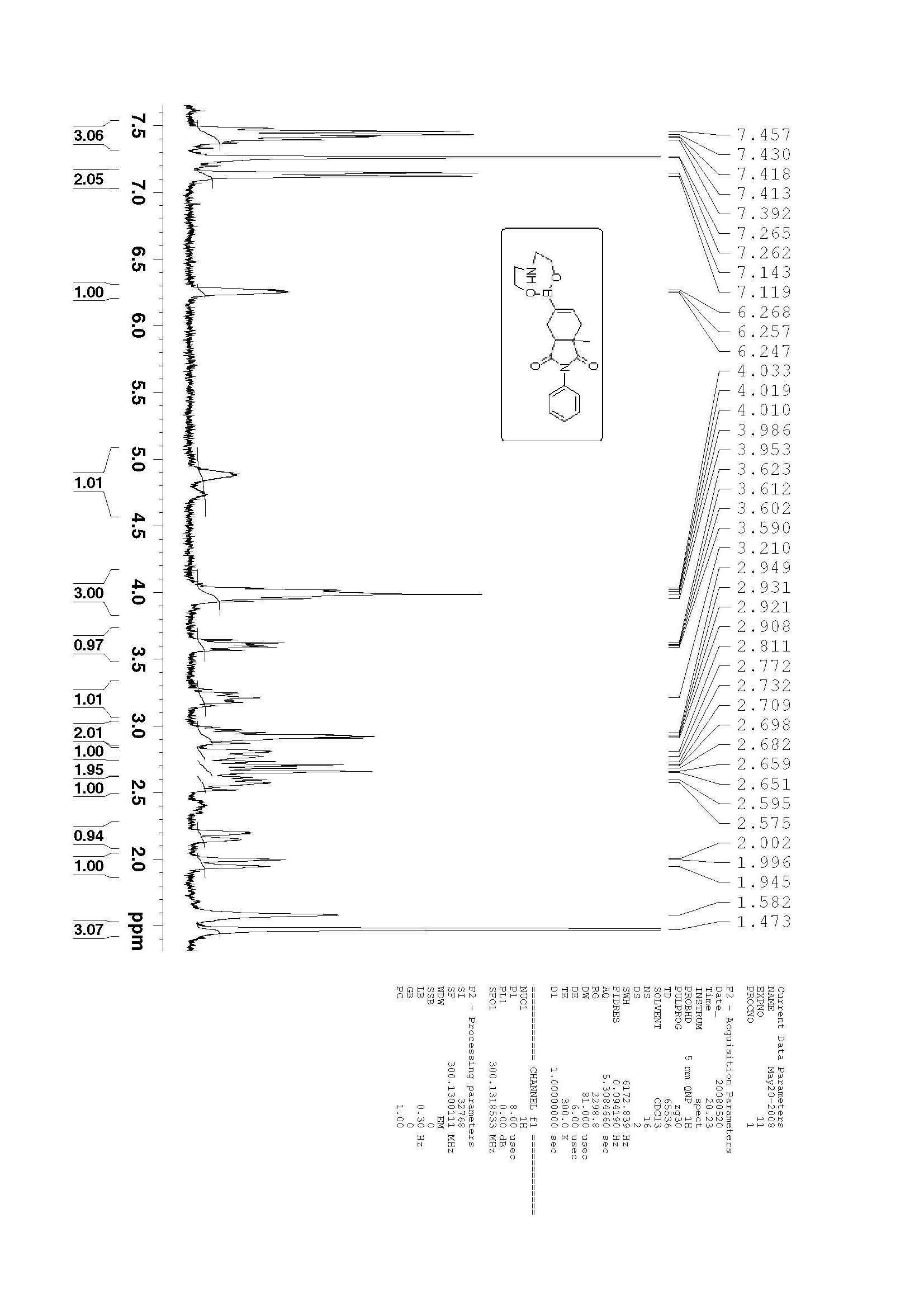


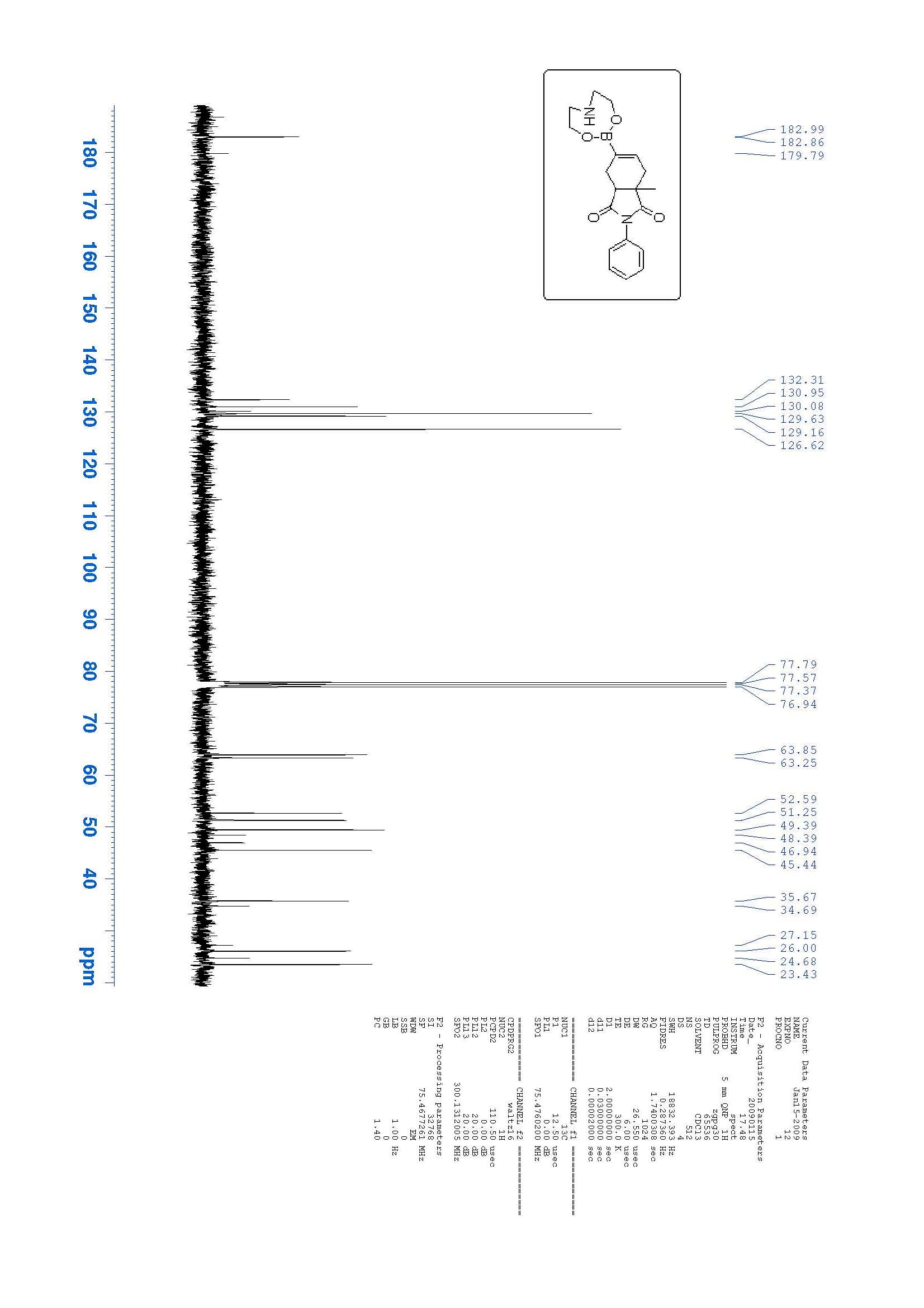


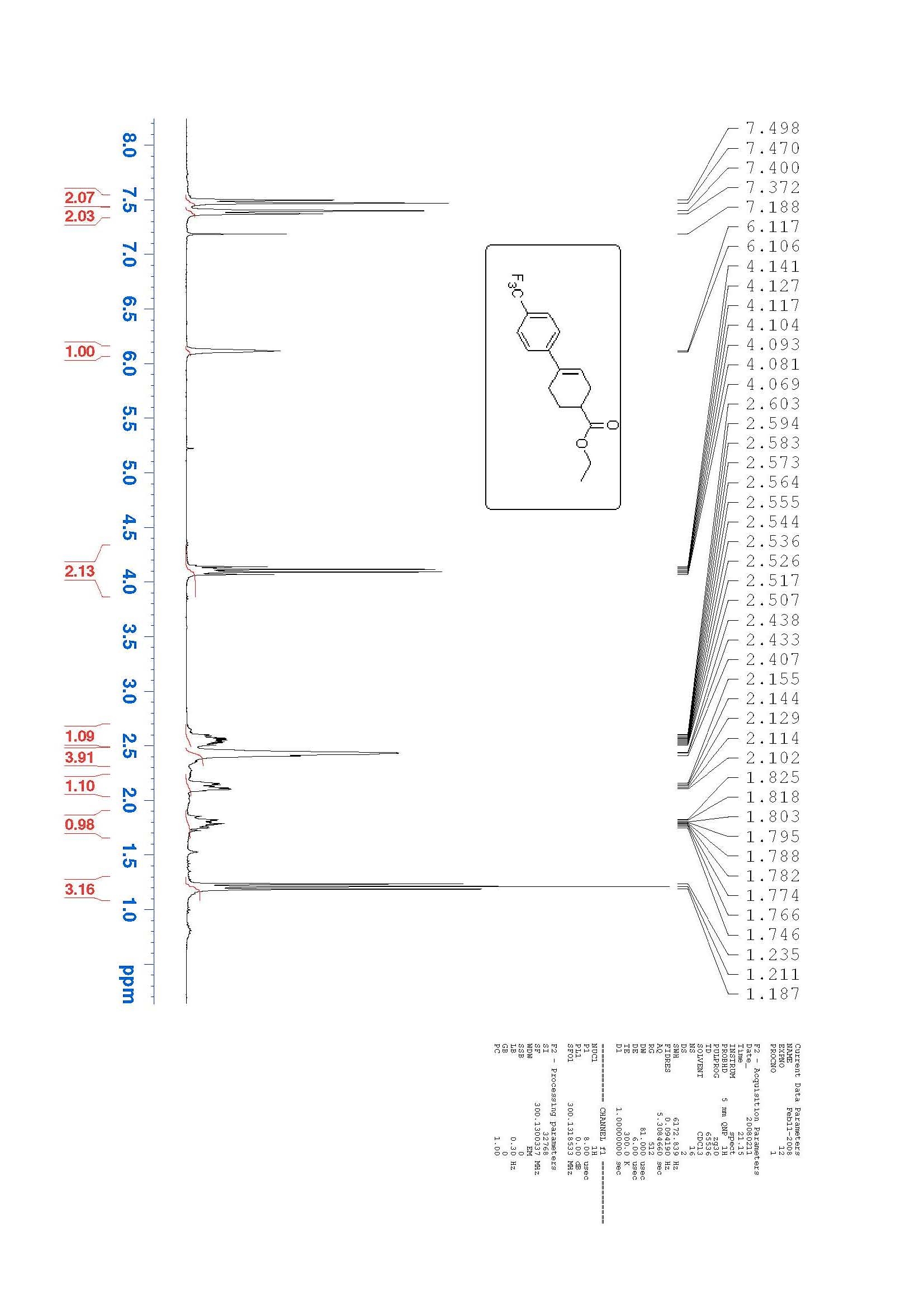


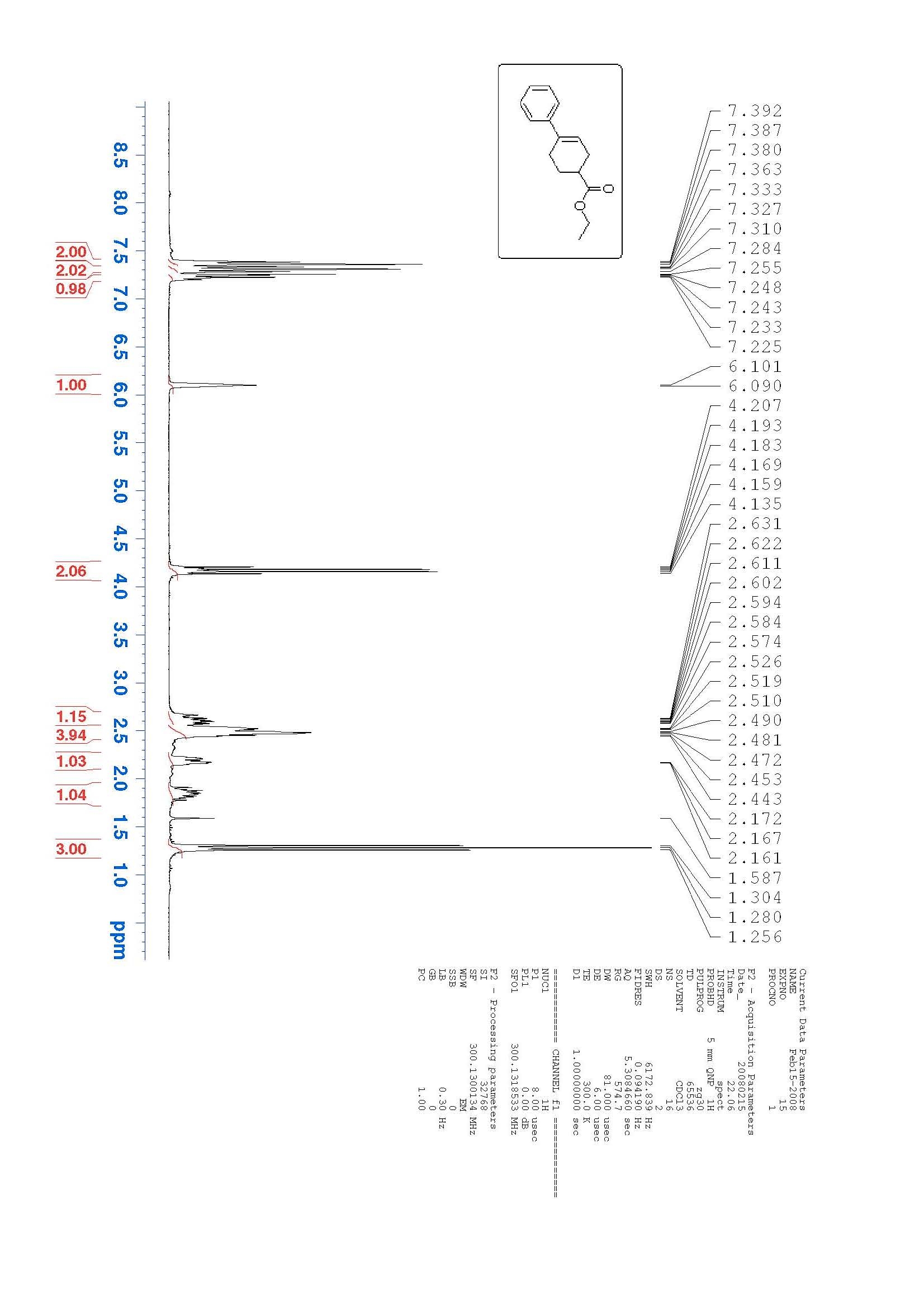


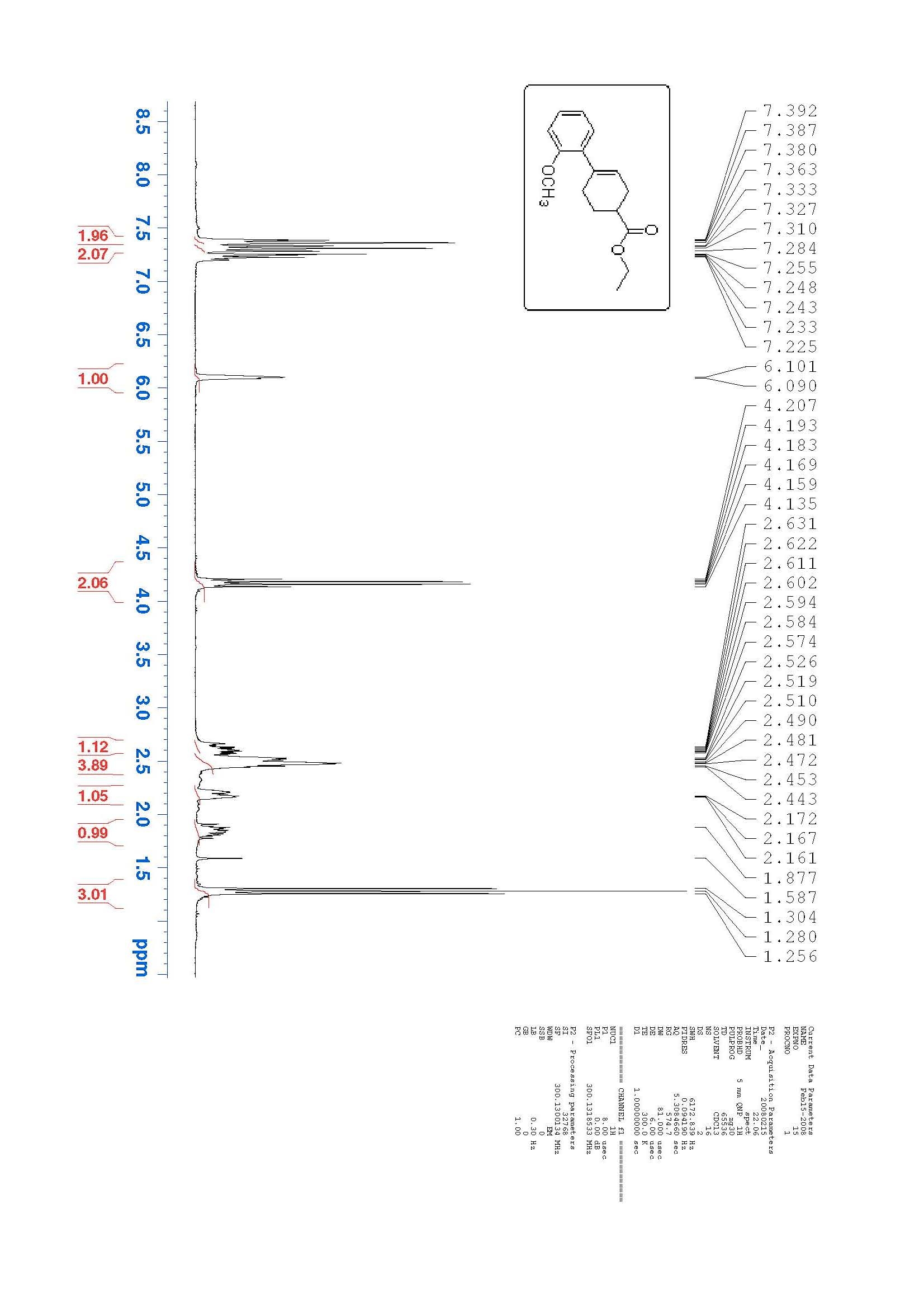


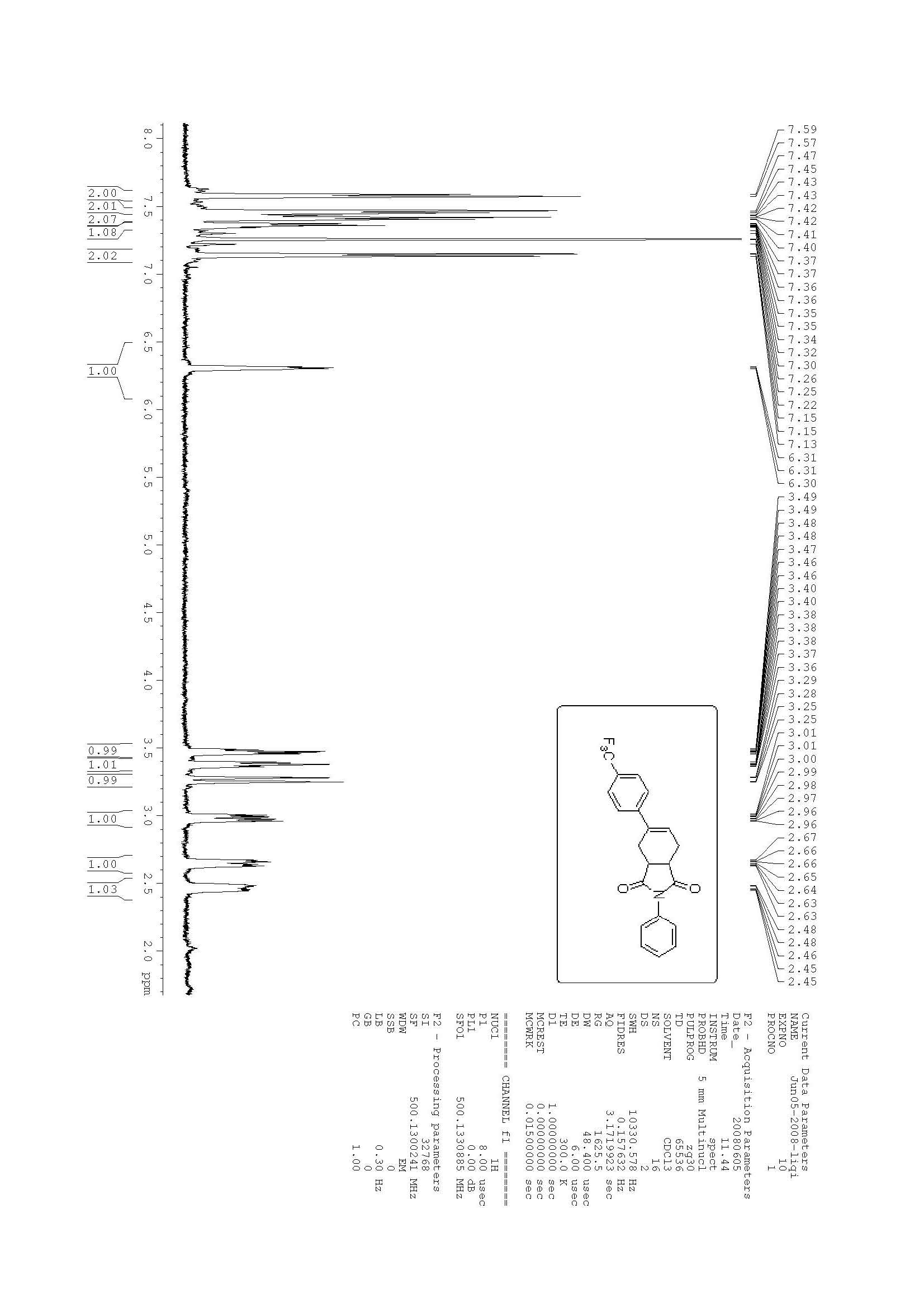


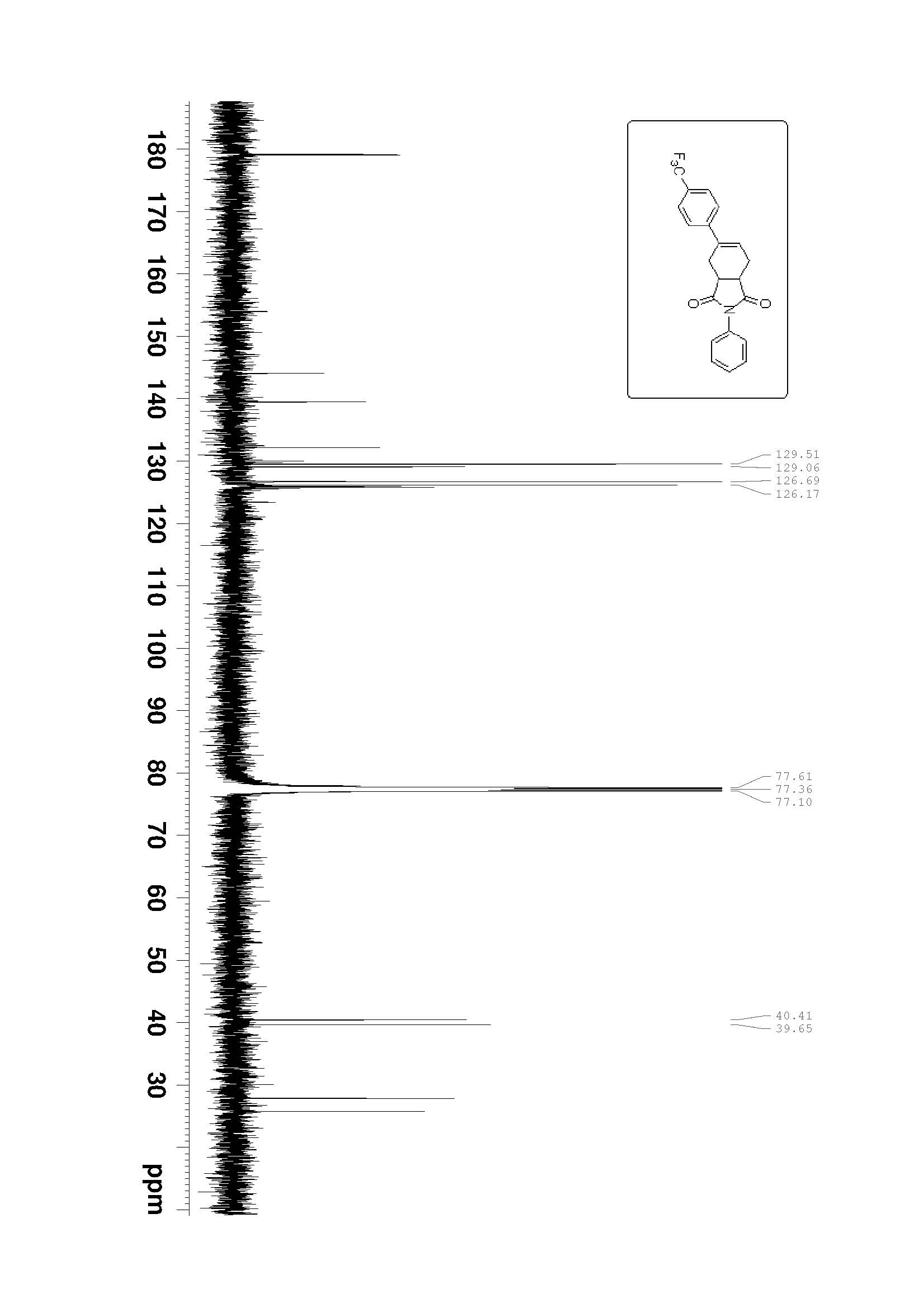


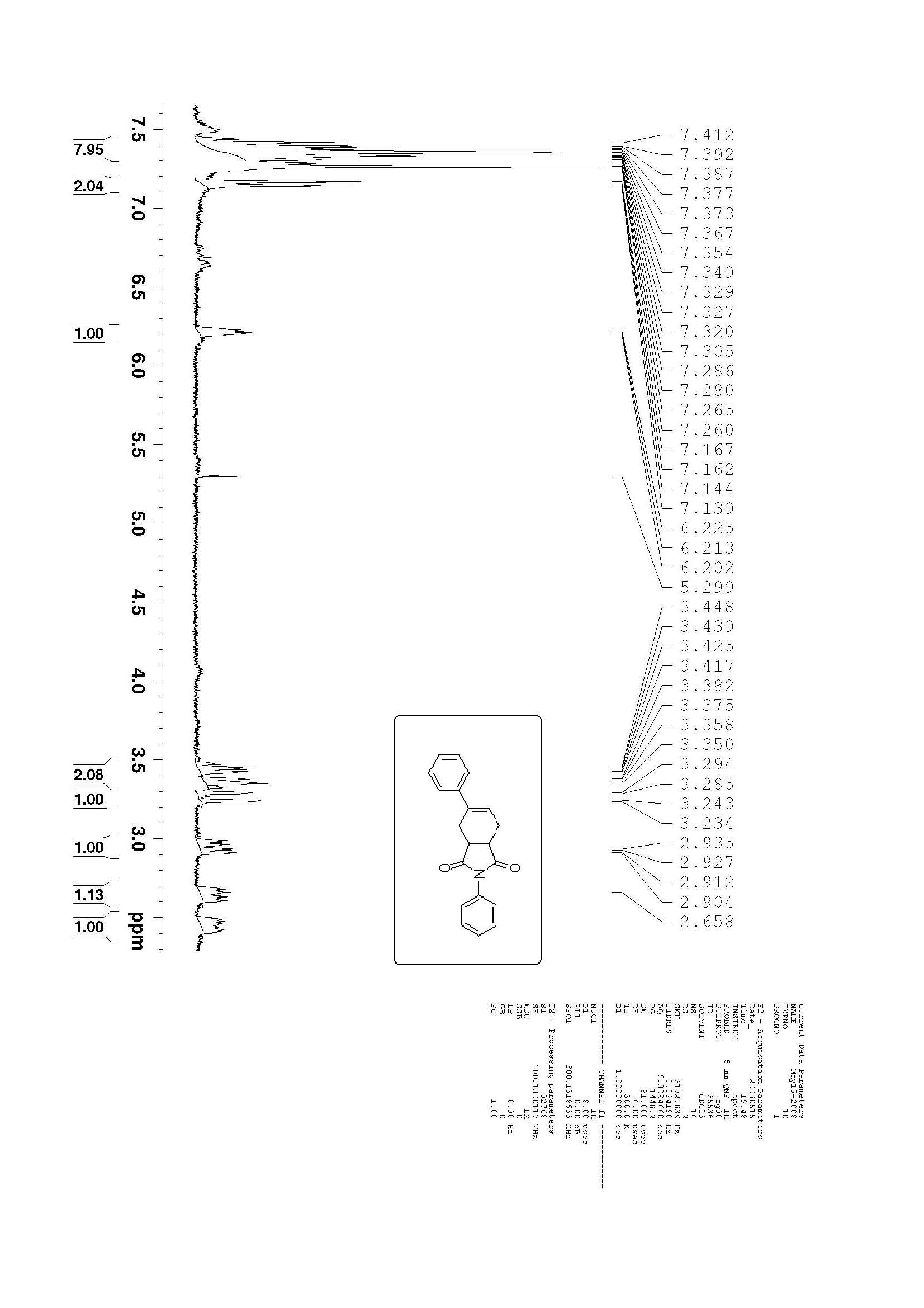


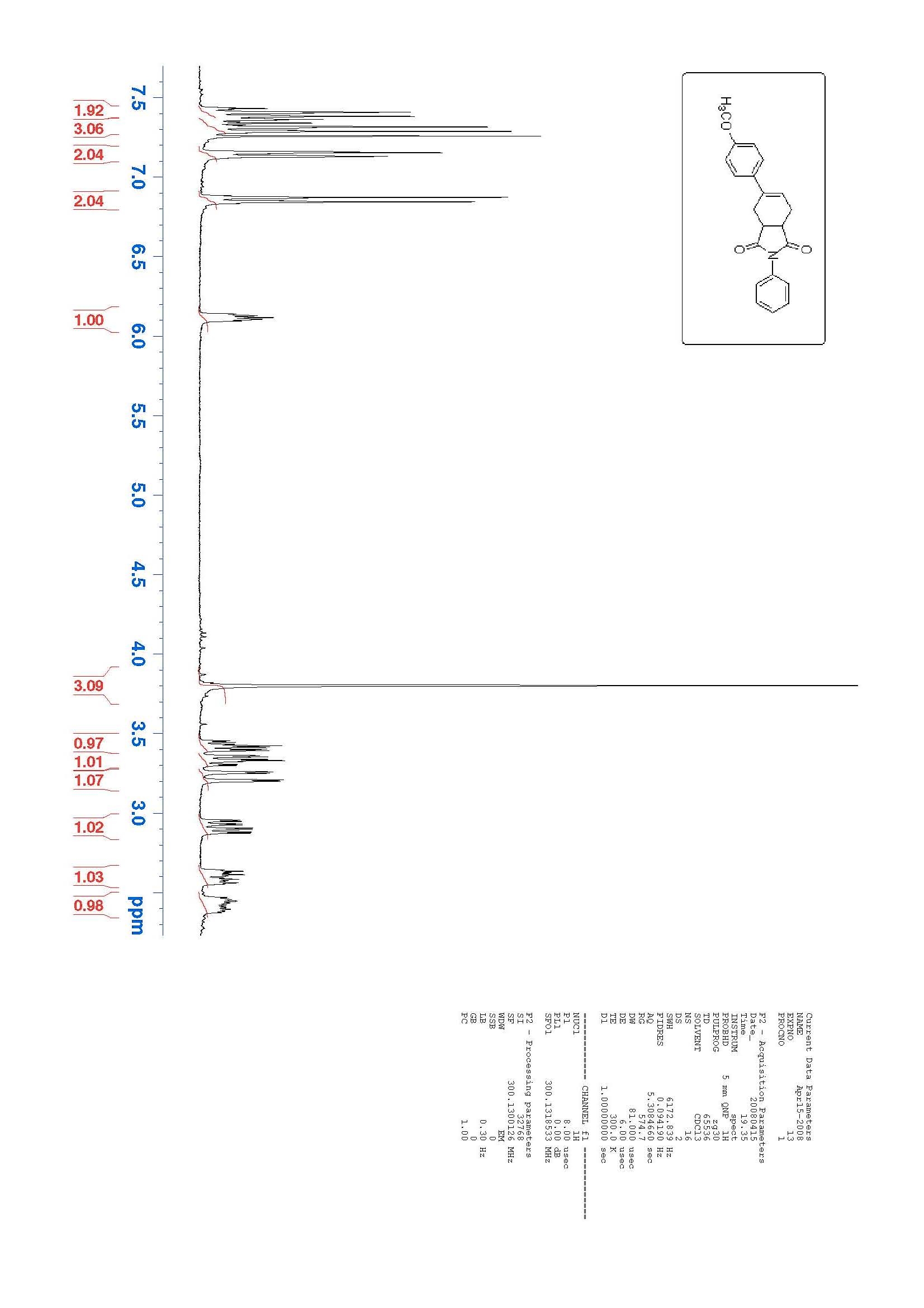


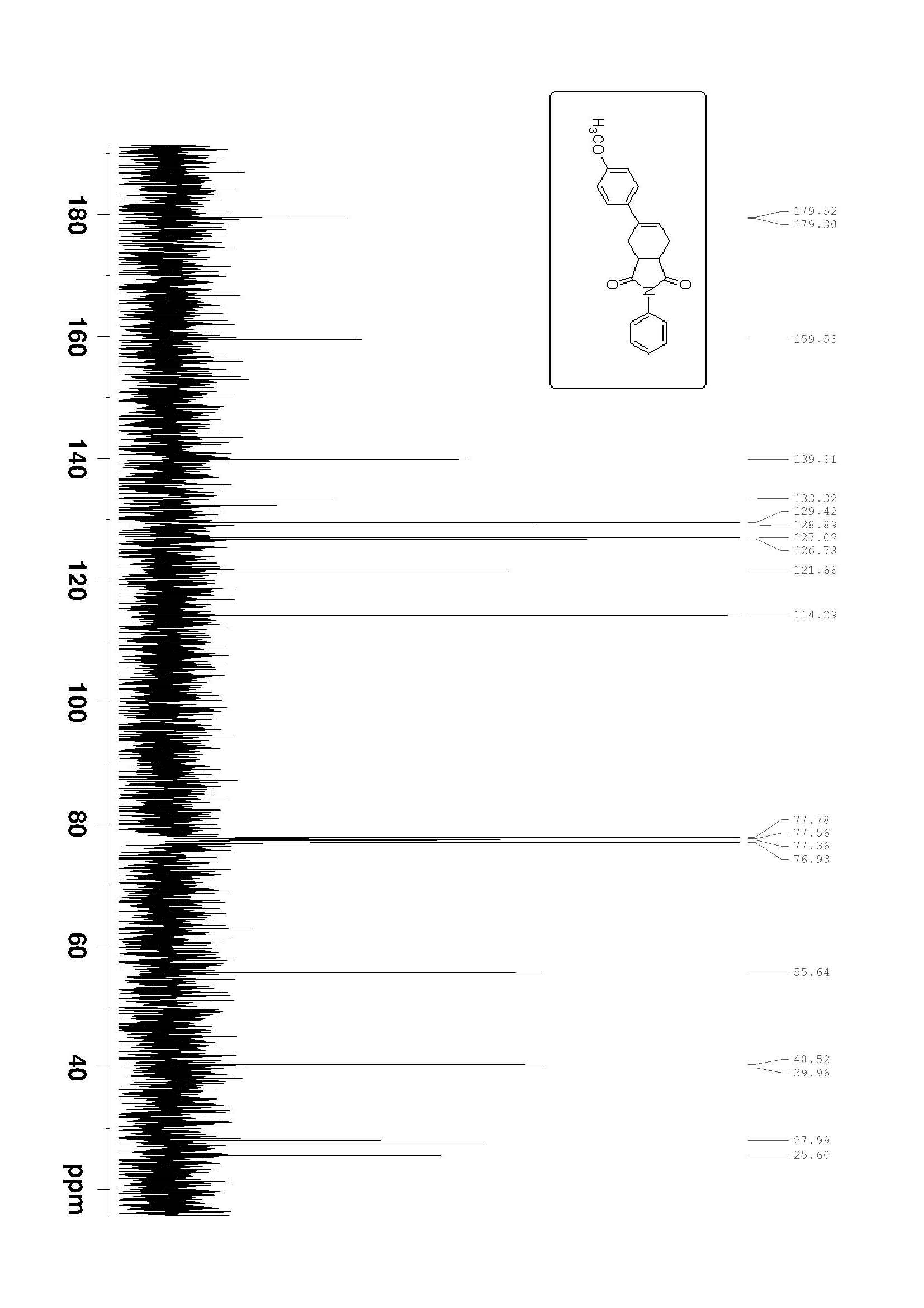


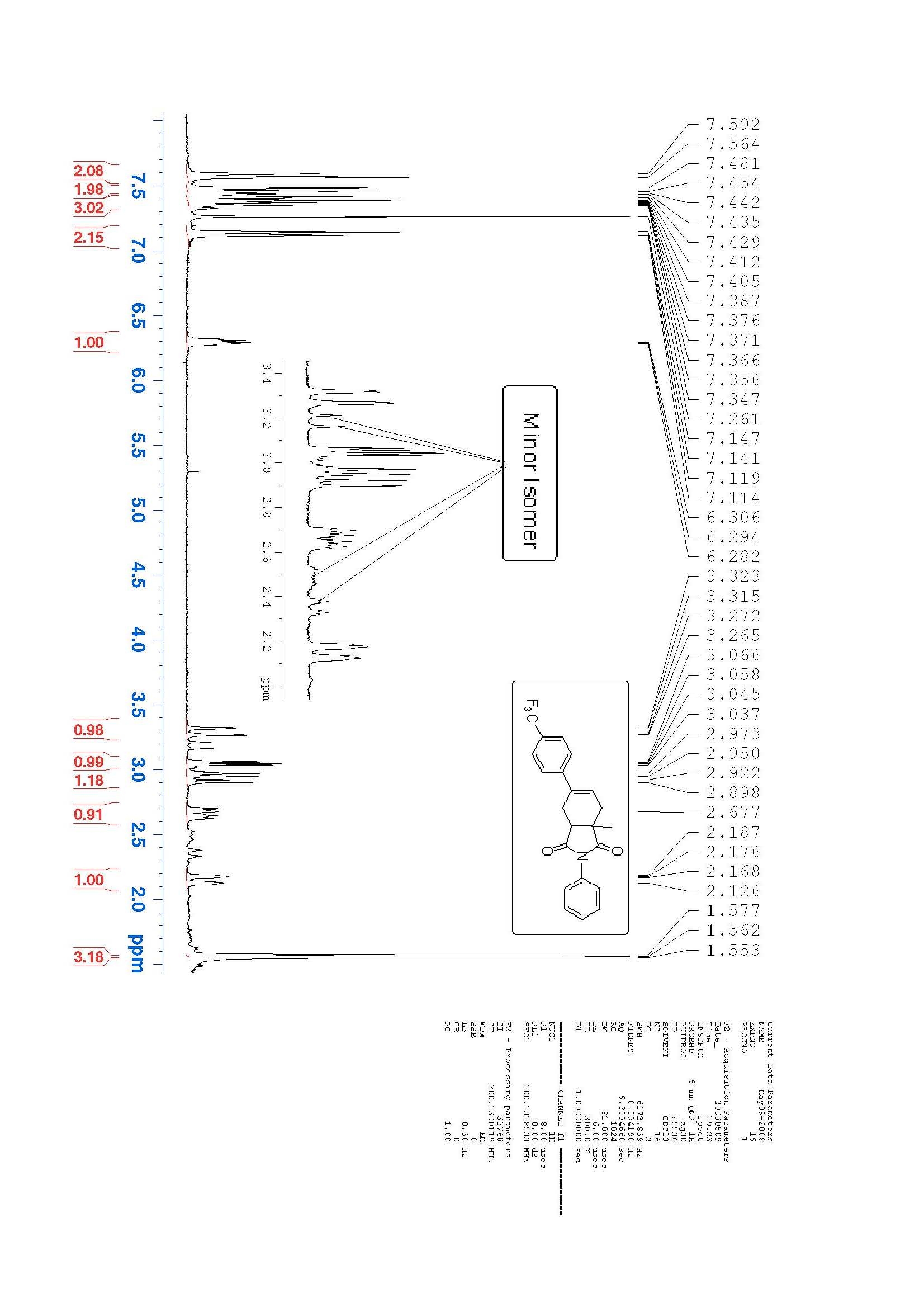


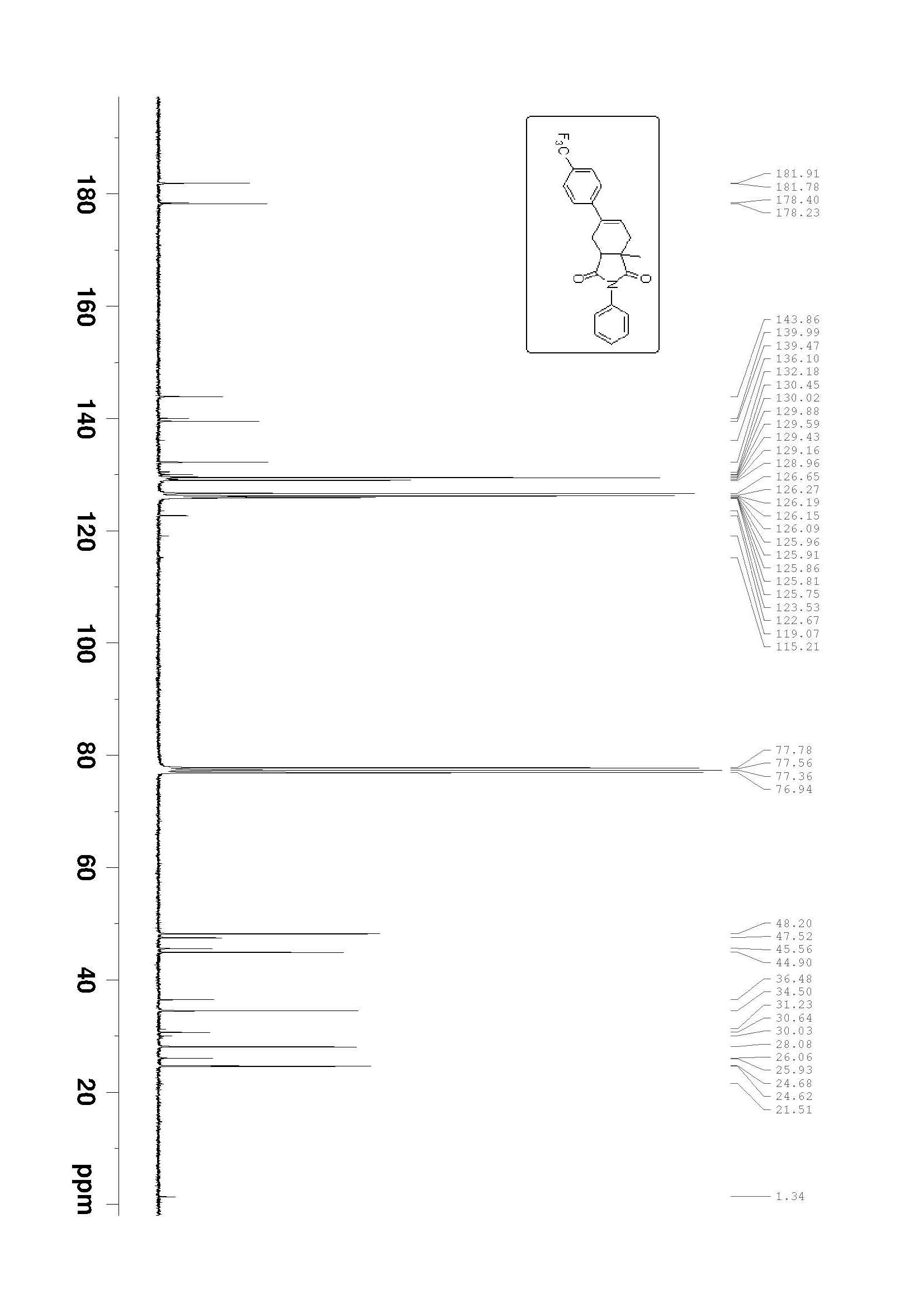


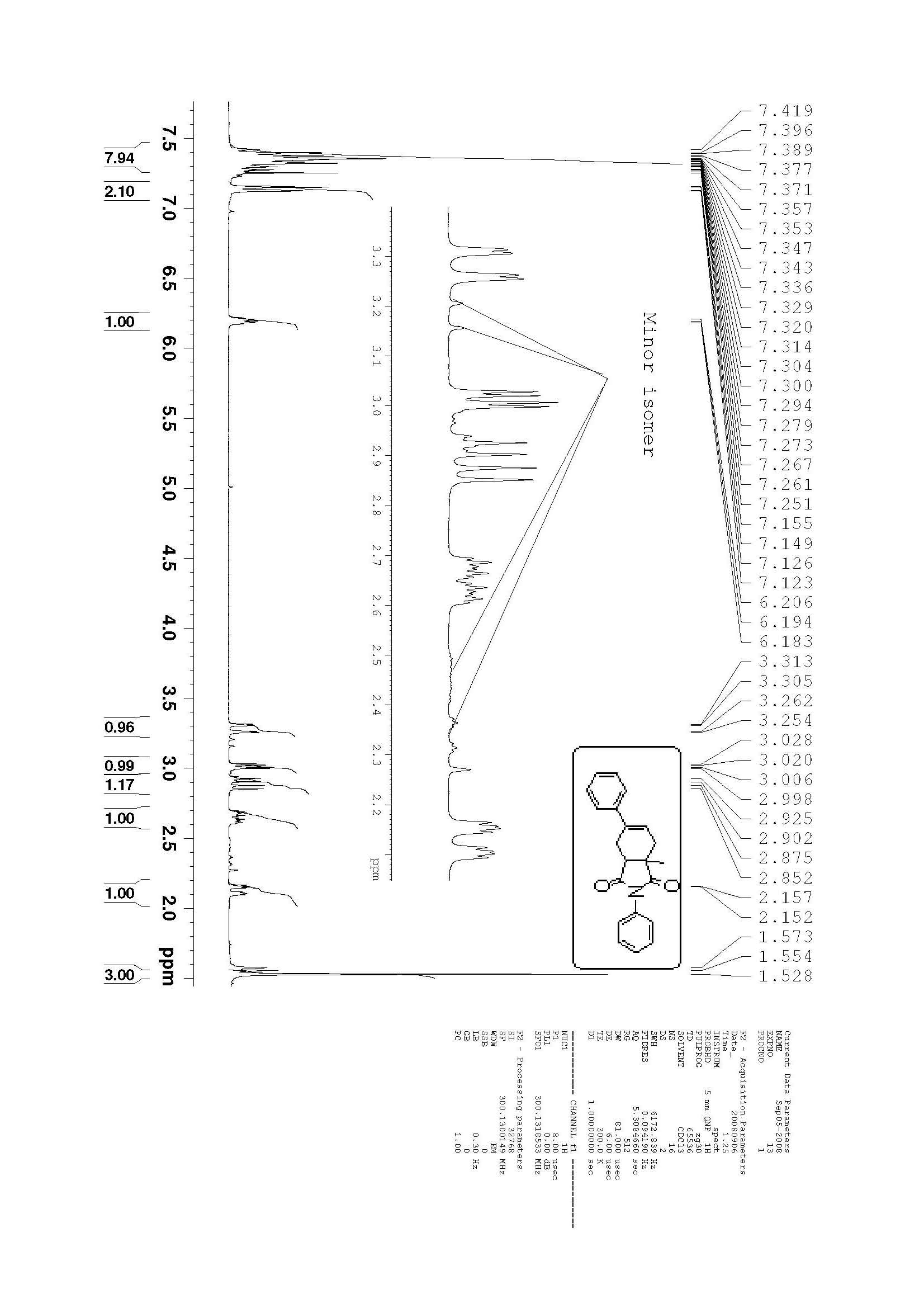


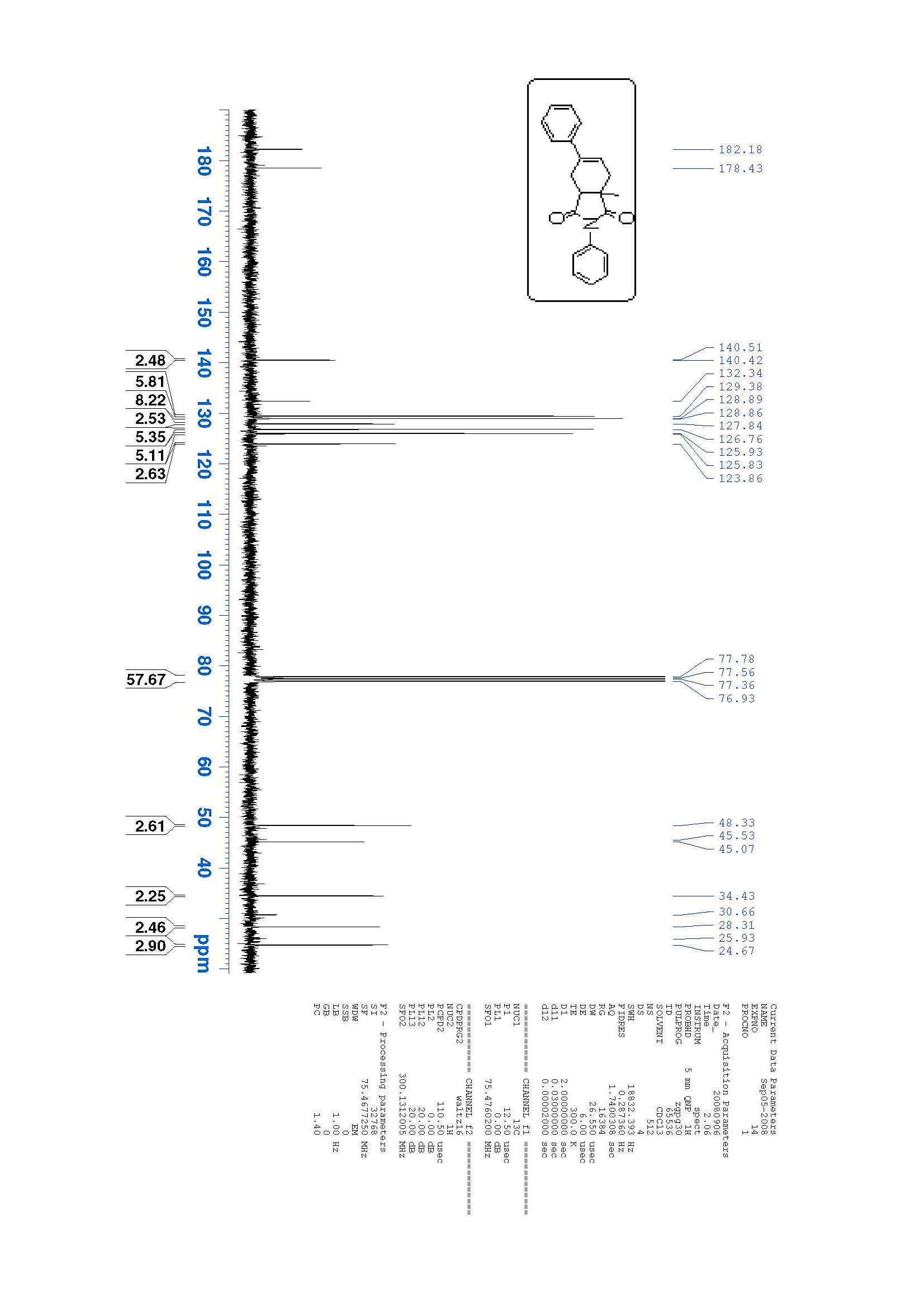


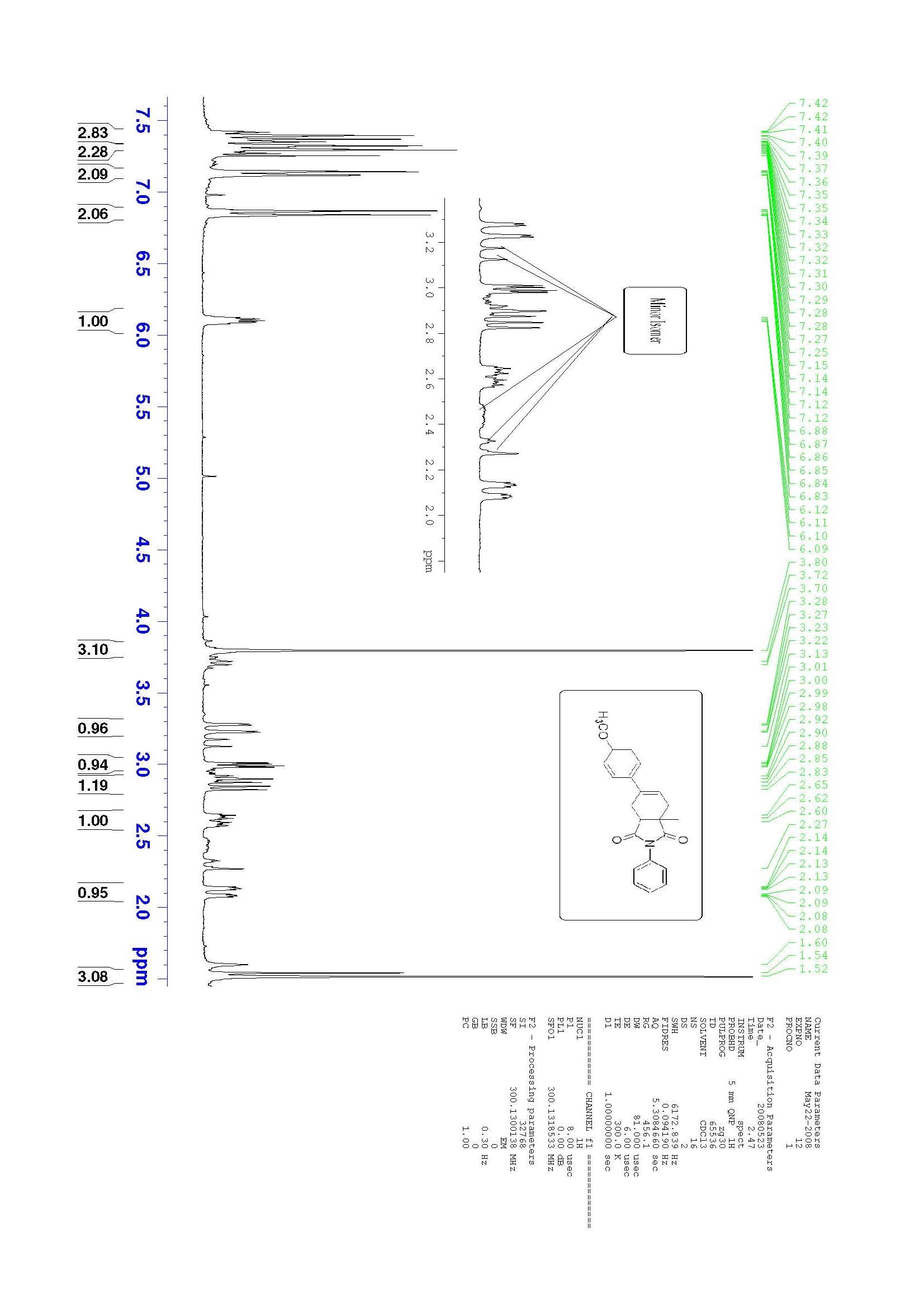


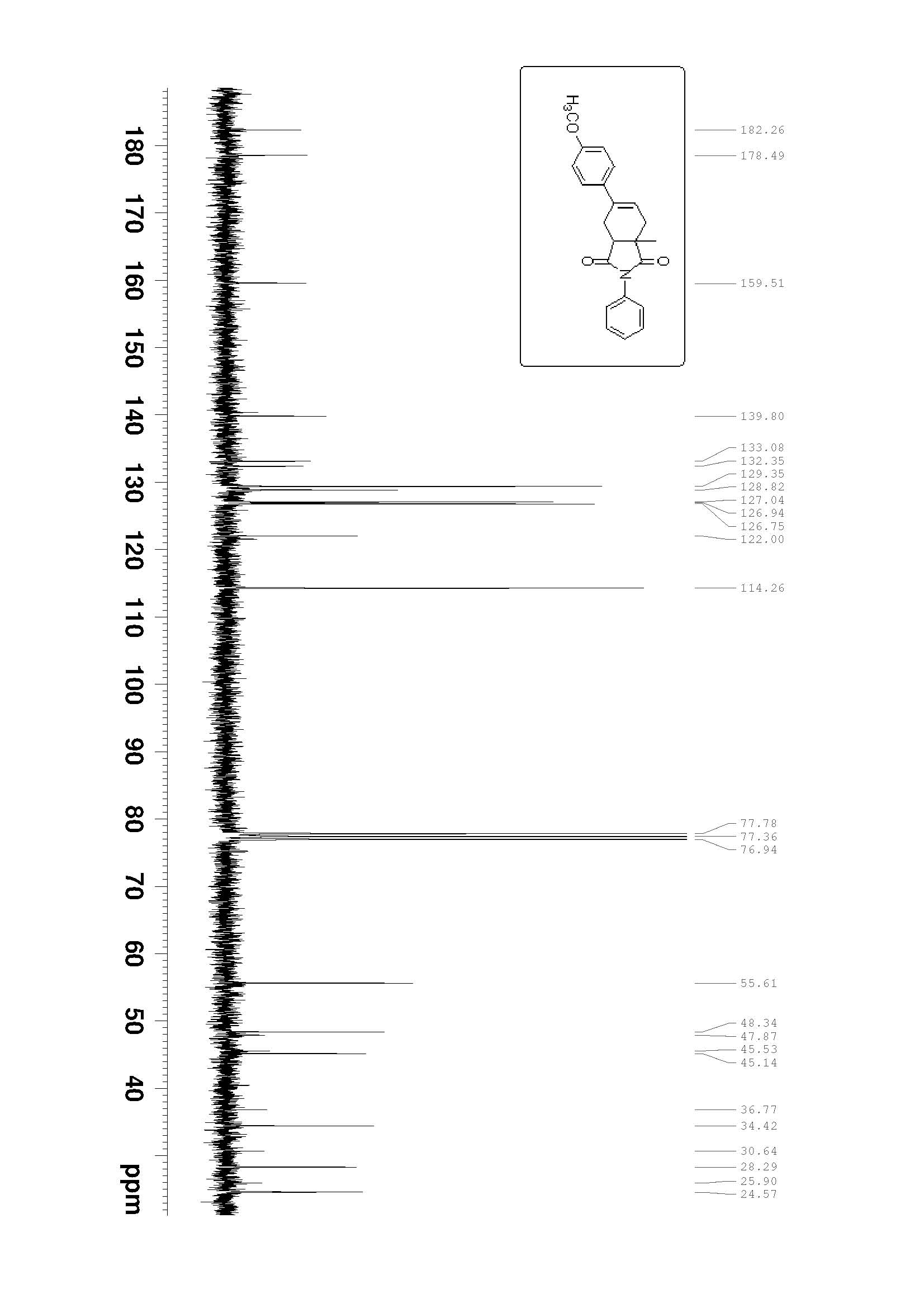

Supplement: File 1 — 1H and 13C NMR spectra of compounds 2–14. [file Beilstein_J_Org_Chem-05-45-s001.doc]
