# Supplementary material for: Preparation and Diels–Alder/cross coupling reactions of a 2-diethanolaminoboron-substituted 1,3-diene
Source: Beilstein J Org Chem. 2009 Sep 21;5:45. doi: 10.3762/bjoc.5.45 (PMC2779738; doi:10.3762/bjoc.5.45)
Supplement: File 2 — Experimental procedures for compounds 4–13. [file Beilstein_J_Org_Chem-05-45-s002.doc]

Preparation and Diels–Alder/cross coupling reactions of a 2-diethanolaminoboron-substituted 1,3-diene

Liqiong Wang, Cynthia S. Day, Marcus W. Wright and Mark E. Welker*

Address: Department of Chemistry, Wake Forest University, P.O. Box 7486, Winston-Salem, NC 27109 (USA)

Email: Mark E. Welker*- welker@wfu.edu

**Keywords:**

cross coupling; Diels–Alder; organoboron

**Supporting Information**

**Experimental section**

**General:** ^1^H NMR were recorded on a Brucker Avance 500 MHz spectrometer on Brucker Avance 300 MHz spectrometer operating at 500.13 MHz and 300.13 MHz respectively. ^13^C NMR were recorded on a Bruker Avance 300 MHz spectrometer and Bruker Avance 500 MHz spectrometer operating at 75.48 MHz and 125.77 MHz respectively. Chemical shifts were reported in parts per million (δ) relative to tetramethylsilane (TMS), or the residual proton resonances in the deuterated solvent: chloroform (CDCl_3_). Coupling constants (*J* values) were reported in Hertz (Hz).

All elemental analyses were performed by Atlantic Microlabs Inc., GA. High resolution mass spectrometric (HRMS) analyses were performed at Caudill Laboratories of the University of North Carolina at Chapel Hill.

All reactions were carried out under an inert atmosphere unless otherwise noted. Flash chromatography was performed using thick walled glass chromatography columns and ultrapure silica gel. THF was purified by passage through a solvent column. Ether and pentane were dried by molecular sieve. Water was deionized and distilled. Absolute ethanol and methanol were used without further purification. Deuterated solvents were purchased from Cambridge Isotope Laboratories and dried over molecular sieves. Magnesium sulfate, magnesium turnings, iodobenzene, 4-iodobenzotrifluorate, 2-iodoanisole, 4-iodoanisole, ethyl acrylate, N-phenylmaleimide and N-phenyl maleimide were purchased from Aldrich Chemical Company and used as received. 2-Chloro-1, 3-butadiene, 50% in xylene (Chloroprene) was purchased from Pfaltz & Bauer and used as received.

Isomer ratios were calculated by NMR (^13^C NMR experiment with inverse gated ^1^H-decoupling [1,2].

Preparation of 1,3-Butadiene-2-diethanolamine borate **2.**

A mixture of magnesium (1.0 g, 41.1 mmol), 1, 2-dibromoethane (0.5 mL), and THF (10 mL) was refluxed under nitrogen for 15 min to activate the magnesium. To the mixture anhydrous zinc chloride (0.6 g) in THF (60 mL) was added and reflux was continued for another 15 min. 2-Chloro-1,3-butadiene (4.9 mL, 25 mmol) (density 0.915 g/mL, 50 % in xylene) and 1,2-dibromoethane (0.95 g, 5 mmol) in THF (30 mL) were added dropwise over a period of 30 min. This addition was controlled so as to bring the mixture into a gentle reflux. The color of the contents changed gradually from grayish white to greenish black. The mixture was heated to reflux for an additional 30 min after completion of the addition. The Grignard reagent thus obtained was immediately added dropwise to a solution of trimethoxyborane (4.25 mL, 38.5 mmol) in THF (25 mL) using a double-ended needle. The addition was controlled in such a way that the internal temperature of the mixture was maintained below –60 °C all the time. After completion of the addition, the solution was allowed to warm to room temperature quickly. The cloudy grey colored reaction mixture was stirred for 1 h . To the resulting mixture at room temperature, 0.5 M HCl solution (100 mL) was added. The reaction mixture was extracted with Et_2_O (2 × 75 mL). The combined colorless clear organic layers were dried over MgSO_4_, and the volatiles were removed by rotovap (30 °C, 20 Torr) to yield diene boronic acid.

The boronic acid was added at once to a solution of diethanolamine (0.8equiv, 22.5 mmol, 8.411g) dissolved in THF (100 mL). Sodium sulfate (8 g) was added and refluxed for 6 hours. At the end of the reaction, the flask was cooled to room temperature. Solid Na_2_SO_4_ was separated from the solution by filtration. The solution was reduced by 50 mL using a rotary evaporator. A cold bath of –30 °C was used to induce crystallization. 4 h later, the solid was filtered and washed with cold chloroform. The product **2** was obtained as white needles following drying under vacuum (2.40 g, 14.4 mmol, 62.4%).

^1^H NMR (300 MHz, CDCl_3_) δ 6.51 (dd, *J* =17.9, 10.9 Hz, 1H-H3), 5.46–5.40 (m, 3H), 4.98 (dd, J=17.9, 1.9 Hz, 1H-H4), 5.18 (s, 1H-H7), 4.05 (m, 2H-H5,8), 3.89 (m, 2H-H5,8), 3.31 (m, 2H-H6,9), 2.76 (m, 2H- H6,9).

^13^C NMR (300, MHz, CDCl_3_) δ 143.6-C3, 124.3-C4, 114.6- C1, 63.4-C5,8, 52.1-C6,9, the signal of carbon (C2) next to a tetravalent boron is generally not observed due to quadrupolar broadening [3].

Elemental anal. calcd for C_8_H_14_BNO_2_: C, 57.53; H, 8.45. Found: 57.06, 8.44.

Preparation of Diels-Alder Product **3.**

Diene **2** (0.167 g, 1 mmol) and dienophile ethyl acrylate (0.700g, 7 mmol) were dissolved in chloroform (15 mL) in a round bottomed flask and refluxed for 6 h. The white product was precipitated with pentane (150 mL) and obtained by vacuum filtration followed by drying under high vacuum, (0.224 g, 0.84 mmol, 84%).

^1^H NMR (300 MHz, CDCl_3_) δ 5.91 (m, 1H), 4.86 (s, 1H), 4.12 (q, *J* = 7.25, 2H), 3.97 (m, 2H), 2.893 (m,2H), 3.224 (m, 2H), 2.79 (m, 2H), 2.48 (m, 1H), 2.23 (m, 2H), 2.11(m, 2H), 1.99 (m, 1H), 1.76 (s, 1H), 1.25 (t, *J* = 7.25, 3H).

^13^C NMR (300 MHz, CDCl_3_) δ **Major isomer:** 176.7, 139.9 (vinyl carbon next to the boron), 126.9, 62.85, 62.81, 60.0, 51.2, 40.1, 39.8, 28.6, 26.4, 25.9, 14.0. **Minor isomer selected resonances**: 176.2, 127.6, 24.7, 24.6

Major isomer : minor isomer = 16.4 : 1

Elemental anal. calcd. for C_13_H_22_BNO_4_: C, 58.45; H, 8.30. Found: 58.17, 8.32.

Preparation of Diels-Alder Product **4.**

Diene **2** (0.167 g, 1 mmol) and dienophile *N*-phenylmaleimide (0.865 g, 5 mmol) were dissolved in chloroform (15 mL) in a round bottom flask at room temperature. After stirring for 15 min., the product **4** was obtained by precipitation as a white powder (0.330g, 0.97mmol, 98%) following addition of pentane (50 mL), vacuum filtration, and drying under vacuum.

^1^H NMR (300 MHz, CDCl_3_) δ 7.49 -7.40 (m, 3H), 7.15-7.11(m, 2H), 6.26 (m, 1H), 4.78 (s, 1H), 4.03-3.94 (m, 3H), 3.61(dt, *J* =9.70, 3.20, 1H), 3.37-3.27 (m, 2H), 3.20 (m, 1H), 2.95 (ddd, *J* =12.3, 12.1, 6.2 Hz, 1H), 2.82-2.54 (m, 4H), 2.31(m, 1H), 2.17 (m, 1H).

^13^C NMR (300 MHz, CDCl_3_) δ 183.7, 180.02, 132.0, 129.8, 129.4, 128.9, 126.3, 63.6, 63.0, 52.4, 51.0, 41.4, 40.3, 26.0, 25.8. The signal of the vinyl carbon next to tetravalent boron was not observed due to quadrupolar broadening [3].

HRMS calcd for C_18_H_21_BN_2_O_4_ [M+H]^+^ = 341.1672, found [M+H]^+^= 341.1673

Preparation of Diels-Alder Product **5.**

Diene **2** (0.167 g, 1 mmol) and dienophile methyl *N*-phenylmaleimide (0.16 g, 1.6 mmol) were dissolved in chloroform(10 mL) in a round bottom flask and refluxed for 6h. The white product was precipitated with pentane (150 mL) and obtained by vacuum filtration followed by drying under high vacuum (0.336 g, 0.95 mmol, 95%).

^1^H NMR (300 MHz, CDCl_3_) δ 7.48-7.37 (m, 3H), 7.14 (s, 1H), 7.12 (s, 1H), 6.26 (m, 1H), 4.88 (s, 1H), 3.99 (m, 3H), 3.61 (m, 1H), 3.21(m, 1H), 2.92 (m, 2H), 2.79 (m, 1H), 2.68 (m, 2H), 2.57 (m, 1H), 2.17 (d, *J* = 15.0 Hz, 1H), 1.97 (d, *J* = 15.0 Hz, 1H), 1.47 (s, 3H).

^13^C NMR (300 MHz, CDCl_3_) δ **Major isomer** : 183.0, 182.9, 132.3, 131.0, 129.6, 129.2, 126.6, 63.9, 63.3, 52.6, 51.8, 49.4, 45.4, 35.7, 26.0, 23.4.

The signal of the vinyl carbon next to tetravalent boron was not observed due to quadrupolar broadening [3].

**Minor isomer** found: 179.8, 130.1, 63.3, 53.0, 48.4, 46.9, 34.7, 27.2, 24.7

Major isomer : minor isomer = 4:1

HRMS calcd for C_19_H_23_BN_2_O_4_ [M+H]^+^=355.1830, found [M+H]^+^= 355.1825

**Suzuki Coupling Reactions.**

General procedure: Boron compounds and iodoaromatic compounds were added to a N_2_ flushed flask with Pd_2_(dba)_3_ and K_2_CO_3_ in acetonitrile and ethanol (30 mL). The mixture was refluxed for 36h and cooled to room temperature. The solution was filtrated through silica gel to remove catalysts. The filtrate was quenched with water (50 mL) and extracted with Et_2_O (4×50 mL). The combined organic layers were dried over MgSO_4_ and volatiles were removed by rotary evaporation. The resulting cross-coupled cycloadduct residue was purified by flash chromatography (ethyl ether: hexane=1:1).

Optimization of conditions: 2% Pd_2_(dba)_3_ [Tris(dibenzylideneacetone)dipalladium (0) ], acetonitrile : ethanol = 5:1, boron cycloadduct : iodoaromatic compounds = 1: 2 , K_2_CO_3_ (3 equiv). reaction time: 36 h.

Preparation of ethyl-4-phenylcyclohex-3-enecarboxylate (**6**)**.**

Following the general procedure, iodobenzene (0.204 g, 1 mmol) and **3** (0.133 g, 0.5 mmol ) were added along with Pd_2_(dba)_3_ (10 mg) and K_2_CO_3_ (0.207 g, 1.5 mmol) to a flask under N_2_ (30 mL acetonitrile and ethanol). The flask was heated and refluxed for 36 h. The resulting brown oily crude product mixture was subjected to flash chromatography to yield the cross-coupled product **6** as a light yellow oil (0.098 g, 0.43 mmol, 85%). ^1^H NMR data was identical to that previously reported [4].

^1^H NMR (300 Hz, CDCl_3_) δ 7.39–7.28 (m, 4H), 7.22 (m, 1H), 6.11–6.08 (m, 1H), 4.169 (q, *J* = 7.3 Hz, 2H), 2.60 (m, 1H), 2.54–2.42 (m, 4H), 2.25–2.14 (m, 1H), 1.84 (m, 1H), 1.27 (t, *J* = 7.3 Hz, 3H).

Major isomer: minor isomer = 17.5:1

Preparation of ethyl 4-[4-(trifluoromethyl)-phenyl]cyclohex-3-enecarboxylate (**7**)**.**

Following the general procedure, 4-iodobenzotrifluoride (0.272 g, 1 mmol) and **3** (0.133 g, 0.5mmol ) were added along with Pd_2_(dba)_3_ (10 mg) and K_2_CO_3_(0.207 g, 1.5 mmol) to a flask under N_2_ (30 mL acetonitrile and ethanol). The flask was heated and refluxed for 36 h. The resulting brown oily crude product mixture was subjected to flash chromatography to yield the cross-coupled product **7** as a light yellow oil (0.144 g, 0.484 mmol, 97%). ^1^H NMR data was identical to that previously reported [4].

^1^H NMR (300 MHz, CDCl_3_) δ 7.48 (d, *J* =8.4 Hz, 1H), 7.38 (d, *J* = 8.4 Hz, 1H), 6.11 (m, 1H), 4.10 (q, *J* = 7.3 Hz, 2H), 2.55 (m, 1H), 2.47–2.39 (m, 4H), 2.13 (m, 1H), 1.79 (m, 1H), 1.209 (t, *J* = 7.3 Hz, 3H).

Major isomer: minor isomer = 18:1

Preparation of ethyl-4-(2-methoxyphenyl) cyclohex-3-enecarboxylate (**8**)**.**

Following the general procedure, 2-iodoanisole (0.234 g, 1 mmol) and **3** (0.133 g, 0.5 mmol ) were added along with Pd_2_(dba)_3_ (10 mg) and K_2_CO_3_(0.207 g, 1.5 mmol) to a flask under N_2_ (30 mL acetonitrile and ethanol). The flask was heated and refluxed for 36 h. The resulting brown oily crude product mixture was subjected to flash chromatography to yield the cross-coupled product **8** as a light yellow oil (0.104 g, 0.40 mmol, 80%). ^1^H NMR data was identical to that previously reported [4].

^1^H NMR (300 Hz, CDCl_3_) δ 7.21 (ddd, *J* = 8.6, 7.4, 1.8 Hz, 1H), 7.103 (dd, *J* = 7.4, 1.8 Hz, 1H), 6.90 (ddd, *J* = 8.6, 7.4, 1.0 Hz, 1H), 6.85 (d, *J* = 8.6 Hz, 1H), 5.75 (m, 1H), 4.166 (q, *J* = 7.3 Hz, 2H), 3.80 (s, 1H), 2.64 (m, 1H), 2.55–2.37 (m, 4H), 2.10 (m, 1H), 1.82 (m, 1H), 1.28 (t, *J* = 7.3 Hz, 3H)

Major isomer: minor isomer = 21.3:1

Preparation of 2,5-diphenyl-3a,4,7,7a-tetrahydro-1*H*-isoindole-1,3(2*H*)-dione (**9**)**.**

Following the general procedure, iodobenzene (0.204 g, 1 mmol) and **4** (0.170 g, 0.5 mmol) were added along with Pd_2_(dba)_3_ (10 mg) and K_2_CO_3_ (0.207 g, 1.5 mmol) to a flask under N_2_ (30 mL acetonitrile and ethanol). The flask was heated and refluxed for 36 h. The resulting brown oily crude product mixture was subjected to flash chromatography to yield the cross-coupled product **9** as a white solid (0.097 g, 0.32 mmol, 64%). ^1^H NMR data was identical to that previously reported [5].

^1^H NMR (300 Hz, CDCl_3_) δ 7.28–7.45 (m, 8H), 7.10–7.20 (m, 2H), 6.15–6.27 (m, 1H), 3.44 (ddd, *J* = 9.5, 6.9, 2.5 Hz, 1H), 3.35 (ddd, *J* = 9.5, 6.9 2.5 Hz, 1H), 3.26 (dd, *J* = 15.5, 2.5 Hz, 1H), 2.95 (ddd, *J* = 15.5, 6.9, 2.5Hz, 1H), 2.64 (ddt, *J* = 15.5, 6.9, 2.5 Hz, 1H), 2.40–2.50 (m, 1H).

Preparation of 2-phenyl-5-[4-(trifluoromethyl)phenyl]-3a,4,7,7a-tetrahydo-1*H*-isoindole-1,3(2*H*)-dione (**10**)**.**

Following the general procedure, 4-iodobenzotrifluoride (0.272 g, 1 mmol) and **4** (0.170 g, 0.5 mmol ) were added along with Pd_2_(dba)_3_ (10 mg) and K_2_CO_3_ (0.207 g, 1.5 mmol) to a flask under N_2_ (30 mL acetonitrile and ethanol). The flask was heated and refluxed for 36 h. The resulting brown oily crude product mixture was subjected to flash chromatography to yield the cross-coupled product **10** as a white solid (0.112 g, 0.3mmol, 60%).

^1^H NMR (300 MHz, CDCl_3_) δ 7.58 (d, *J* = 8.0 Hz, 2H), 7.46 (d, *J* = 8.0 Hz, 2H), 7.42 (t, *J* =8.0 Hz, 2H), 7.3 (t, *J* = 8.0 Hz, 1H), 7.14 (d, *J* = 8.0 Hz, 2H), 6.31 (m, 1H), 3.48 (ddd, *J* = 9.1, 7.4, 2.5 Hz, 1H), 3.38 (ddd, *J* = 9.1, 7.4, 2.5 Hz, 1H), 3.26 (dd, *J* = 15.5, 2.7 Hz, 1H), 2.98 (ddd, *J* = 15.5, 7.1, 2.7 Hz, 1H), 2.65 (ddt, *J* = 15.5, 7.1, 2.7 Hz, 1H), 2.43–2.50 (m, 1H).

^13^C NMR (300 MHz, CDCl_3_) δ 179.2, 179.0, 144.0, 139.5, 132.1, 129.9 ( ^2^*J*_C–F_ = 32.6 Hz), 129.5, 129.1, 126.7,126.3 ( ^1^*J*_C–F_ = 273.1 Hz), 126.2, 125.9( ^3^*J*_C–F_ = 3.8 Hz), 125.8, 40.4, 39.7, 27.8, 25.7.

HRMS calcd for C_21_H_16_F_3_NO_2_ [M+H]^+^ = 372.1211, found [M+H]^+^ = 372.1211.

Preparation of 5-(4-methoxyphenyl)-2-phenyl-3a,4,7,7a-tetrahydro-1*H*-isoindole-1,3(2*H*)-dione (**11**)**.**

Following the general procedure, 4-iodoanisole (0.234 g, 1 mmol) and **4** (0.170 g, 0.5 mmol ) were added along with Pd_2_(dba)_3_ (10 mg) and K_2_CO_3_ (0.207 g, 1.5 mmol) to a flask under N_2_ (30 mL acetonitrile and ethanol). The flask was heated and refluxed for 36 h. The resulting brown oily crude product mixture was subjected to flash chromatography to yield the cross-coupled product **11** as a white solid (0.099 g, 0.3 mmol, 60%). ^1^H NMR data was identical to that previously reported [5].

^1^H NMR (300 Hz, CDCl3) δ 7.43–7.28 (m,5H), 7.15 (d, *J* = 7.6 Hz, 2H), 6.86 (d, *J* = 8.7 Hz, 2H), 6.12 (m, 1H), 3.80 (s, 3H), 3.42 (ddd, *J* = 9.3, 7.0, 2.6 Hz, 1H), 3.33 (ddd, *J* = 9.3, 7.0, 2.5 Hz,1H), 3.26 (dd, *J* = 15.5, 2.5 Hz, 1H), 2.95 (ddd, *J* = 15.5, 6.9, 2.5 Hz, 1H), 2.64 (ddt, *J* = 15.5, 6.9, 2.5 Hz, 1H), 2.40−2.50 (m, 1H).

Preparation of 3a-methyl-2,6-diphenyl-3a,4,7,7a-tetrahydro-1*H*-isoindole-1,3(2*H*)-dione (**12**)**.**

Followed the general procedure, 4-iodobenzene (0.204 g, 1 mmol) and **5** (0.178 g, 0.5 mmol ) were added along with Pd_2_(dba)_3_ (10 mg) and K_2_CO_3_ (0.207 g, 1.5 mmol) to a flask under N_2_ (30 mL acetonitrile and ethanol). The flask was heated and refluxed for 36 h. The resulting brown oily crude product mixture was subjected to flash chromatography to yield the cross-coupled product **12** as a white solid (0.0887 g, 0.28 mmol, 58%).

^1^H NMR (300 MHz, CDCl_3_) δ **Major isomer:** 7.42–7.23 (m, 8H), 7.16 (m, 1H), 7.13 (m, 1H), 6.19 (m, 1H), 3.283 (ddd, *J* = 15.30, 2.4 Hz, 1H), 3.01 (dd, *J* = 6.6, 2.4 Hz, 1H), 2.886 (dd, *J* = 6.6 Hz, 1H), 2.65 (ddt, *J* = 15.30, 6.6, 2.4 Hz, 1H), 2.12 (ddd, *J* = 15.30, 3.7, 2.4 Hz, 1H), 1.53 (s, 3H).

**Minor isomer found:** 3.18 (d, *J* = 15.3), 2.46 (d, *J* = 15.3), 2.34 (d, *J* = 15.3)

^13^C NMR (300 MHz, CDCl_3_) δ 182.2, 178.4, 140.5, 140.4, 132.3, 129.4, 128.9, 128.8, 127.8, 126.8, 125.9, 125.8, 123.9, 48.3, 45.5, 34.4, 28.3, 25.9.

HRMS calcd for C_21_H_19_NO_2_ [M+H]^+^= 318.1494, found [M+H]^+^ = 318.1494.

Major isomer: minor isomer = 3:1

Preparation of 3a-methyl-2-phenyl-6-[4-(trifluoromethyl)phenyl]-3a,4,7,7a-tetrahydro-1*H*-isoindole-1,3(2*H*)-dione (**13**)**.**

Following the general procedure, 4-iodobenzotrifluoride (0.272 g, 1 mmol) and **5** (0.177 g, 0.5 mmol ) were added along with Pd_2_(dba)_3_ (10 mg) and K_2_CO_3_ (0.207 g, 1.5 mmol) to a flask under N_2_ (30 mL acetonitrile and ethanol). The flask was heated and refluxed for 36 h. The resulting brown oily crude product mixture was subjected to flash chromatography to yield the cross-coupled product **13** as a white solid (0.135 g, 0.35 mmol, 70%).

^1^H NMR (300 MHz, CDCl_3_) δ **Major isomer:** 7.58 (d, *J* = 8.0 Hz, 2H), 7.47 (d, *J* = 8.0 Hz, 2H), 7.44–7.32 (m, 3H), 7.14–7.11 (m, 2H), 6.29 (m, 1H), 3.29 (dd, *J* = 15.2, 2.5 Hz, 1H), 3.05 (dd, *J* = 6.50, 2.5 Hz, 1H), 2.93 (m, 1H), 2.68 (ddt, *J* = 15.2, 6.5, 2.5 Hz, 1H), 2.15 (m, 1H), 1.56 (s, 3H); **Minor isomer** found: 3.188 (d, *J* = 15.2), 2.35 (dt, *J* = 15.2, 2.5 Hz)

^13^C NMR APT (300 MHz, CDCl_3_) δ **Major isomer:** 181.9, 178.4, 143.9, 139.5, 132.2, 129.8, 129.6, 129.4 ( ^2^*J*_C–F_ = 33.3 Hz) , 126.7, 126.3, 126.1, 125.9( ^1^*J*_C–F_ = 273.3 Hz), 125.5 ( ^3^*J*_C–F_ =4Hz), 48.2, 44.9, 34.5, 28.1, 24.7 **Minor isomer** found: 180.0, 176.5, 140.0, 47.5, 45.6, 36.5, 30.6, 26.1.

HRMS calcd for C_22_H_18_F_3_NO_2_ [M+H]^+^= 386.1368, found [M+H]^+^= 386.1368.

Major isomer: minor isomer = 3:1

Preparation of 6-(4-methoxyphenyl)-3a-methyl-2-phenyl-3a,4,7,7a-tetrahydro-1*H*-isoindole-1,3(2*H*)-dione (**14**)**.**

Following the general procedure, 4-iodoanisole (0.234 g, 1 mmol) and **5** (0.178 g, 0.5 mmol) were added along with Pd_2_(dba)_3_ (10 mg) and K_2_CO_3_ (0.207 g, 1.5 mmol) to a flask under N_2_ (30 mL acetonitrile and ethanol). The flask was heated and refluxed for 36 h. The resulting brown oily crude product mixture was subjected to flash chromatography to yield the cross-coupled product as a white solid (0.134 g, 0.39 mmol, 78%).

^1^H NMR (300 MHz, CDCl_3_) δ **Major isomer:** 7.38 (d, *J* = 7.5 Hz, 2H), 7.31 (d, *J* = 8.7 Hz, 2H), 7.26 (m, 1H), 7.13 (d, *J* = 7.5 Hz, 2H), 6.85 (d, *J* = 8.8 Hz, 2H), 6.1 (m, 1H), 3.80 (s, 3H), 3.25 (dd, *J* = 15.4, 2.4 Hz, 1H), 2.99 (dd, *J* = 6.5, 2.4 Hz, 1H), 2.86 (dd, *J* = 15.4, 6.5 Hz, 1H), 2.61 (ddt, *J* = 15.4, 6.5, 2.4 Hz, 1H), 2.16 (dd, *J* = 15.4, 2.4 Hz, 1H), 1.50 (s, 3H); **Minor isomer selected resonances**: 3.15 (d, *J* = 15.4), 2.44 (m), 2.30 (m).

^13^C NMR (300 MHz, CDCl_3_) δ 182.3, 178.5, 159.5, 139.8, 133.1, 132.4, 129.4, 128.8, 127.0, 126.8, 122.0, 114.3, 55.6, 48.4, 45.1, 36.7, 30.6, 25.9.

Elemental anal. calcd for C_22_H_21_NO_3_: C, 76.06; H, 6.09. Found: 76.34, 6.31.

Major isomer: minor isomer = 3:1

1. Berger, S. a. B., Siegmar, 200 and More NMR Experiments. **1998**, 318-320.

2. Robert M. Silverstein, F. X. W. a. D. J. K., Spectrometric Identification of Organic Compounds,Seventh edition. 213-214.

3. Darses, S. M., Guillaume; Genet, Jean-Pierre., Potassium organotrifluoroborates. New partners in palladium-catalyzed cross-coupling reactions. . *European Journal of Organic Chemistry* **1999,** 8, 1875-1883.

4. De, S., Preparation and Tandem Reactions of 2-Trifluoroborate Substituted Dienes. *Ph.D Dissertation of Wake Forest University Graduate School of Arts & Sciences* **2007**.

5. Ramakrishna R. Pidaparthi, M. E. Welker., Cynthia S. Day, and Marcus W. Wright, Preparation of 2-Trialkylsiloxy- Substituted 1,3-Dienes and Their Diels−Alder/Cross-Coupling Reactions. *Organic Letters* **2007,** 9, (9), 1623-1626.

**X-Ray crystallographic** **comments**

X-ray crystallographic data has been deposited with the CCDC and allocated deposition number CCDC 736603. The asymmetric unit in crystalline C_8_H_14_NO_2_B – CHCl_3_ contains one C_8_H_14_NO_2_B molecule and one chloroform molecule of crystallization. All displacement ellipsoids are drawn at the 50% probability level.

Brief experimental description to be included as text or as a footnote at time of publication

Colorless plate-shaped crystals of C_8_H_14_NO_2_B – CHCl_3_ are, at 203(2) K, orthorhombic, space group Pna2_1_ – C (No. 33) [1] with a = 19.587(3) Å, b = 7.742(1) Å, c = 9.104(1) Å, V = 1380.6(3) Å^3^ and *Z* = 4 formula units {d_calcd_ = 1.378 g/cm^3^; μ_a_(MoK$\overline{\alpha}$) = 0.648 mm^-1^}. A full hemisphere of diffracted intensities (1968 30-second frames with a ω scan width of 0.30°) was measured for a single domain specimen using graphite-monochromated MoK$\overline{\alpha}$ radiation (λ = 0.71073 Å) on a Bruker SMART APEX CCD Single Crystal Diffraction System [2]. X-rays were provided by a fine-focus sealed x-ray tube operated at 50 kV and 30 mA.

Lattice constants were determined with the Bruker SAINT software package using peak centers for 1895 reflections having 8.07°≤ 2θ ≤ 40.42°. A total of 10091 integrated absorption-corrected reflection intensities having 2θ((MoK$\overline{\alpha}$)< 49.98° were produced using the Bruker program SAINT [3]; 2411 of these were unique and gave R_int_ = 0.040 with a coverage which was 99.5% complete. The Bruker software package SHELXTL was used to solve the structure using “direct methods” techniques. All stages of weighted full-matrix least-squares refinement were conducted using F_o_^2^ data with the SHELXTL Version 6.12 software package [4].

The final structural model incorporated anisotropic thermal parameters for all nonhydrogen atoms and isotropic thermal parameters for all hydrogen atoms. All hydrogen atoms on the diene (H1A, H1B, H3, H4A and H4B) as well as the amine hydrogen atom (H1N) were located in a difference Fourier and included in the structural model as independent isotropic atoms whose parameters were allowed to vary in least-squares refinement cycles. The remaining hydrogen atoms were included in the structural model as fixed atoms (using idealized sp^3^-hybridized geometry and C‑H bond lengths of 0.98 – 0.99 Å) "riding" on their respective carbon atoms. The isotropic thermal parameters for these hydrogen atoms were fixed at a value 1.2 times the equivalent isotropic thermal parameter of the carbon atom to which they are covalently bonded. A total of 169 parameters were refined using one restraint and 2411 data. Final agreement factors at convergence are: R_1_(unweighted, based on F) = 0.045 for 2083 independent “observed” reflections having 2θ(MoK$\overline{\alpha}$)< 49.98° and I>2σ(I); R_1_(unweighted, based on F) = 0.053 and wR_2_(weighted, based on F^2^) = 0.096 for all 2411 independent reflections having 2θ(MoK$\overline{\alpha}$)< 49.98°. The largest shift/s.u. was 0.000 in the final refinement cycle. The final difference map had maxima and minima of 0.258 and -0.167 e^-^/Å^3^, respectively. The absolute structure was determined by refinement of the Flack parameter x [5]; x refined to a final value of 0.03(8) .

**Acknowledgment**

The authors thank the National Science Foundation (grant CHE-0234489) for funds to purchase the x-ray instrument and computers.

**References**

1. International Tables for Crystallography, Vol A, 4^th^ ed., Kluwer Academic Publishers: Boston (1996).
2. Data Collection: SMART (Version 5.628) (2002). Bruker-AXS, 5465 E. Cheryl Parkway, Madison, WI 53711-5373 USA.
3. Data Reduction: SAINT (Version 6.45) (2003). Bruker-AXS, 5465 E. Cheryl Parkway, Madison, WI 53711-5373, USA and Sheldrick, G. M. (2006). SADABS (Version 2006/3). University of G$\text{ö}$ttingen, Germany.
4. G. M. Sheldrick (2001). SHELXTL (Version 6.12). Bruker-AXS, 5465 E. Cheryl Parkway, Madison, WI 53711-5373 USA.
5. Flack, H. D. (1983). *Acta Cryst.* **A39**, 876-881.
